# Supplementary material for: Involvement of Target of Rapamycin (TOR) Signaling in the Regulation of Crosstalk between Ribosomal Protein Small Subunit 6 Kinase-1 (RPS6K-1) and Ribosomal Proteins
Source: Plants (Basel). 2023 Jan 1;12(1):176. doi: 10.3390/plants12010176 (PMC9824793; doi:10.3390/plants12010176)
Supplement: Supplementary file 1 [file plants-12-00176-s001.zip › plants-2095424-supplementary.pdf]

Involvement of Target of Rapamycin (TOR) signaling in the regulation of crosstalk between Ribosomal Protein Small Subunit 6 Kinase-1 (RPS6K-1) and ribosomal proteins

Achala Bakshi[1, 3*], Mazahar Moin[1, 5], Meher B. Gayatri[2], Aramati B. M. Reddy[2], Raju Datla[3*], M. S. Madhav[1, 4*], P. B. Kirti[5]

[1]Indian Institute of Rice Research, Rajendranagar, Hyderabad-500030, Telangana, India
[2]Department of Animal Biology, University of Hyderabad, Hyderabad-500046, Telangana, India
[3]Global Institute for Food Security, Saskatoon, SK, S7N 0W9, Canada
[4]Central Tobacco Research Institute, Rajahmundry-533105, Andhra Pradesh, India
[5]Agri Biotech Foundation, PJTS Agricultural University Campus, Rajendranagar, Hyderabad-500030, Telangana, India

[*] Correspondence to:
Achala Bakshi: achala.bakshi@gifs.ca;
Raju Datla: raju.datla@gifs.ca;
M. S. Madhav: sheshu24@gmail.com

Contact details of authors:
Mazahar Moin: moinmazahar@gmail.com;
Meher B. Gayatri: meher.gaya3@gmail.com;
Aramati B. M. Reddy: binduaramati@gmail.com;
P. B. Kirti; pbkirti@uohyd.ac.in, pbkirti@gmail.com

Key words: Target of Rapamycin; Ribosomal proteins large subunit genes; Ribosomal proteins small subunit genes

Abbreviations: *TOR*, *Target of Rapamycin*; *TOR*-OE, *TOR* overexpressing; RPs, Ribosomal Proteins; RPL, Ribosomal protein large subunit; RPS, Ribosomal protein small subunit; WT, Wild Type; DAG, Days after Germination; S6K1, Ribosomal Protein Small Subunit 6 Kinase1

```
OsRPS6A         MKFNIANPTTGCQKKLEIDDDQKLRAFFDKRISQEVSGDALGEEFKGYVFKIMGGCDKQG
OsRPS6B         MKFNIANPTTGCQKKLEIDDDQKLRAFYDKRISQEVSGDALGEEFKGYVFKIMGGCDKQG
AtRPS6A         MKFNVANPTTGCQKKLEIDDDQKLRAFYDKRISQEVSGDALGEEFKGYVFKIKGGCDKQG
AtRPS6B         MKFNVANPTTGCQKKLEIDDDQKLRAFFDKRLSQEVSGDALGEEFKGYVFKIMGGCDKQG
                ****:**********************:***:******************** *******

OsRPS6A         FPMKQGVLTAGRVRLLLHRGTPCFRGYGRRDGERRRKSVRGCIVSQDLSVINLVIVKKGE
OsRPS6B         FPMKQGVLTSGRVRLLLHRGTPCFRGYGRRDGERRRKSVRGCIVSQDLSVINLVIVKKGD
AtRPS6A         FPMKQGVLTPGRVRLLLHRGTPCFRGHGRRTGERRRKSVRGCIVSPDLSVLNLVIVKKGE
AtRPS6B         FPMKQGVLTPGRVRLLLHRGTPCFRGHGRRTGERRRKSVRGCIVSPDLSVLNLVIVKKGV
                *********.****************:*** ************** ****:********

OsRPS6A         NDLPGLTDTEKPRMRGPKRASKIRKLFNLSKDDDVRKYVNTYRRTFTTKNGKKVSKAPKI
OsRPS6B         NDLPGLTDTEKPRMRGPKRASKIRKLFNLAKDDDVRKYVNTYRRTFTTKNGKKVSKAPKI
AtRPS6A         NDLPGLTDTEKPRMRGPKRASKIRKLFNLKKEDDVRTYVNTYRRKFTNKKGKEVSKAPKI
AtRPS6B         SDLPGLTDTEKPRMRGPKRASKIRKLFNLGKEDDVRKYVNTYRRTFTNKKGKKVSKAPKI
                .**************************** *:****.*******.**.*:**:*******

OsRPS6A         QRLVTPLTLQRKRARIADKKKRIAKKKSEAAEYQKLLAQRLKEQRERRSESLAKRRSKLS
OsRPS6B         QRLVTPLTLQRKRARIAQKKQRIAKKKSEAAEYQKLLAQRLKEQRERRSESLAKRRSKLS
AtRPS6A         QRLVTPLTLQRKRARIADKKKKIAKANSDAADYQKLLASRLKEQRDRRSESLAKKRSRLS
AtRPS6B         QRLVTPLTLQRKRARIADKKKRIAKANSDAADYQKLLASRLKEQRDRRSESLAKKRSRLS
                *****************:**::*** :*:**:******.******:********:**:**

OsRPS6A         SAAKAAATTA
OsRPS6B         AATTA-----
AtRPS6A         SAAAKPSVTA
AtRPS6B         SAPAKPVAA-
                :*.
```

Figure S1. Multiple sequence alignment of RPL and RPS proteins identifies conserved Ser/ Thr phosphorylation sites

Sequence alignment of RPS6A/B proteins of *Arabidopsis thaliana* and *Oryzasativa*ssp. *japonica* were aligned using CLUSTAL Omega to identify the conserved sites for phosphorylation by AGC kinase family kinases specifically at Ser/ Thr residues.The Arabidopsis and rice RPS6 showed highest similarity of more than 90%. The sequence alignment of rice (*Oryza sativa* ssp. *japonica*) RPS6A/B proteins from ssp. *japonica* shared highest similarity with *Arabidopsis thaliana* RPS6A/B proteins in their Ser/Thr phosphorylation sites at similar amino acid positions (Thr9,Thr 10,Thr 69,Thr 81,Thr 127,Thr 129,Thr 161,Thr 165,Thr 167,Thr 168,Thr 185,Thr 188,Thr 248,Thr 249, Ser33 , Ser 37 , Ser 98, Ser 105, Ser 109, Ser 141, Ser 150, Ser 175, Ser 208, Ser 229, Ser 231, Ser 237, Ser 240, Ser 241; Supplementary Figures S1–S6), The OsRPL6 protein has twenty five Ser/ Thr phosphorylation sites for various AGC kinases (PKA, PKB, PKC, PKG and RSK)

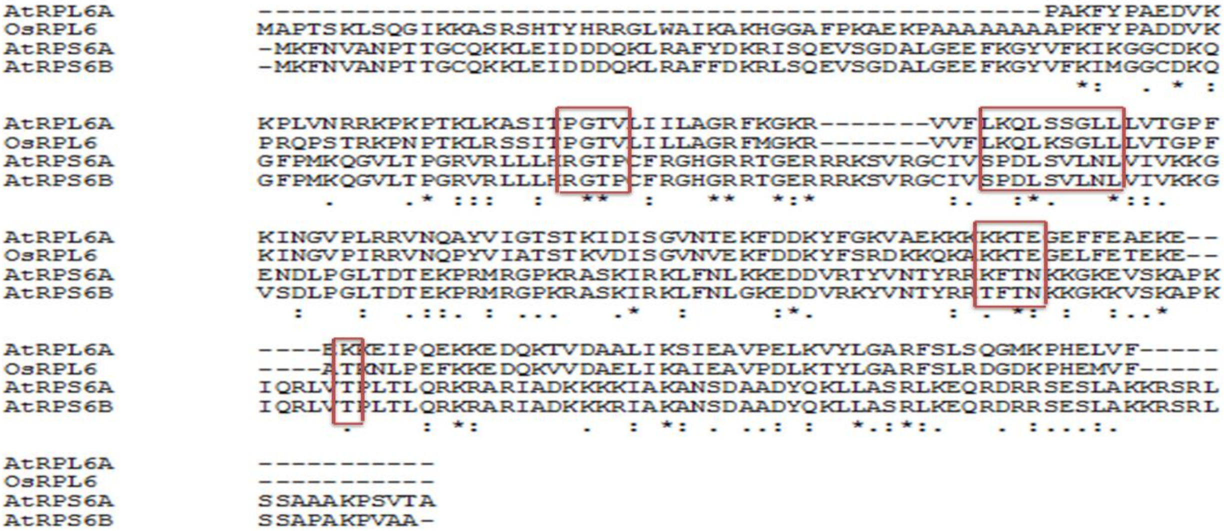

Figure S2. Sequence alignment of RPS6A/B and RPL6 proteins of *Arabidopsis thaliana* and *Oryzasativa* ssp. *japonica*. The conserved Ser/Thr phosphorylation peptide sites are highlighted in red boxes.

```
AtRPS6A             MKFNVANPTTGCQKKLEIDDDQKLRAFYDKRISQEVSGDALGEEFKGYVFKIKGGCDKQG
AtRPS6B             MKFNVANPTTGCQKKLEIDDDQKLRAFFDKRLSQEVSGDALGEEFKGYVFKIMGGCDKQG
AtRPL18e/L15P       ---------------MGIDLIAGGKSKKTKRTAPKSDDVYLKLLVKLYRFLVR----RSN
AtRL18C             ---------------MGIDLIAGGKSKKTKRTAPKSDDVYLKLLVKLYRFLVR----RSN
AtRPL18e/L15        ------------------------------------------------------------
OsRL18A             ---------------------MGAFRFHQYQVVGRGLPTPTDEHPKIYR-----------

AtRPS6A             FPMKQGVLTPGRVRLLLHRGTPCFRGHGRRTGERRRKSVRGCIVSPDLSVLNLVIVKKGE
AtRPS6B             FPMKQGVLTPGRVRLLLHRGTPCFRGHGRRTGERRRKSVRGCIVSPDLSVLNLVIVKKGV
AtRPL18e/L15P       SNFNAVILKRLFMSKVNKAPLSLSRLVEPMTGKDDKIAVLVGTITDDLRVHEIPAMK---
AtRL18C             SNFNAVILKRLFMSKVNKAPLSLSRLVEPMTGKDDKIAVLVGTITDDLRVHEIPAMK---
AtRPL18e/L15        ------------MSKVNKAPLSLSRLVEPMTGKDDKIAVLVGTITDDLRVHEIPAMK---
OsRL18A             --MKLWATNEVRAKSKFWYFLRKLKKVKKSNGQILAINEIFEKNPTTIKNYGIWLRYQS-
                                            :     .*:           .  :    :

AtRPS6A             NDLPGLTDTEKPRMRGPKRASKIRKLFNLKKEDDVRTYVNTYRRKFTNKKGKEVSKAPKI
AtRPS6B             SDLPGLTDTEKPRMRGPKRASKIRKLFNLGKEDDVRKYVNTYRRTFTNKKGKKVSKAPKI
AtRPL18e/L15P       --VTALRFTERARARIEKAGGECLTFDQLALR-----------------------APLG
AtRL18C             --VTALRFTERARARIEKAGGECLTFDQLALR-----------------------APLG
AtRPL18e/L15        --VTALRFTERARARIEKAGGECLTFDQLALR-----------------------APLG
OsRL18A             --RTGYHNMYKEYRDTTLNGAVEQMYTEMASR------------------------HRV
                       ..     :        ..      ::  .

AtRPS6A             QRLVTPLTLQRKRARIADKKKKIAKANSDAADYQKLLASRLKEQRDRRSESLAKKRSRLS
AtRPS6B             QRLVTPLTLQRKRARIADKKKRIAKANSDAADYQKLLASRLKEQRDRRSESLAKKRSRLS
AtRPL18e/L15P       QNTVLLRGPKNSREAVKHFGPAPGVPHSNTKPYVRHKGRKFEKARGKRKSRGFKV-----
AtRL18C             QNTVLLRGPKNSREAVKHFGPAPGVPHSNTKPYVRHKGRKFEKARGKRKSRGFKV-----
AtRPL18e/L15        QNTVLLRGPKNSREAVKHFGPAPGVPHSNTKPYVRHKGRKFEKARGKRKSRGFKV-----
OsRL18A             RFPCIQIIKTATVHFKLCKRDNTKQFHKSDIKFPLVYRKVRPPTRKLKTTFKASRPNLFM
                    :          .              :..  :           *  :.    .

AtRPS6A             SAAAKPSVTA
AtRPS6B             SAPAKPVAA-
AtRPL18e/L15P       ----------
AtRL18C             ----------
AtRPL18e/L15        ----------
OsRL18A             ----------
```

Figure S3. Alignment of RPS6A/B and RPL18 proteins of *Arabidopsis thaliana* and *Oryza sativa* ssp. *japonica*.

```
AtRPS6A             MKFNVANPTTGCQKKLEIDDDQKLRAFYDKRISQEVSGDALGEEFKGYVFKIKGGCDKQG
AtRPS6B             MKFNVANPTTGCQKKLEIDDDQKLRAFFDKRLSQEVSGDALGEEFKGYVFKIMGGCDKQG
OsRPL23-1           ---------------------------MSKRGRGGSAGNKFRMSLGLPVAATVNCADNTG
OsRPL23-2           ---------------------------MSKRGRGGSAGNKFRMSLGLPVAATVNCADNTG
AtRPL23aA           --------------------------MSPAKVDTTKKADPKAKALKAAKAVKSGQAFKKK
AtRPL23aB           --------------------------MSPAKVDVTKKADPKAKALKAAKAVKSGQIVKKP
                                                  :      .:     :        .   :

AtRPS6A             FPMKQGVLTPGRVRILLHRGTPCFRGHGRRTGERRRKSVRGCIVSPDLSVLNLVIVKKGE
AtRPS6B             FPMKQGVLTPGRVRILLHRGTPCFRGHGRRTGERRRKSVRGCIVSPDLSVLNLVIVKKGV
OsRPL23-1           AKNLYIISVKGIKGRLNRLPSACVG------------------------DMVMATVKKGK
OsRPL23-2           AKNLYIISVKGIKGRLNRLPSACVG------------------------DMVMATVKKGK
AtRPL23aA           DKKIRTKVTFHRPKTLTKPRTGKYP--------------------------KISATPRNK
AtRPL23aB           AKKIRTKVTFHRPKTLTVPRKPKYP--------------------------KISATPRNK
                            .      *    .                               :  . :.

AtRPS6A             NDLPGLTDTEKPRMRGPKRASKIRKLFNLKKEDDVRTYVNTYRRKFTNKKGKEVSKAPKI
AtRPS6B             SDLPGLTDTEKPRMRGPKRASKIRKLFNLGKEDDVRKYVNTYRRTFTNKKGKKVSKAPKI
OsRPL23-1           PDLR----------KKVMPAVIVRQRKPWRRKDGVYMYFEGIIHR---------------
OsRPL23-2           PDLR----------KKVMPAVIVRQRKPWRRKDGVYMYFEDNAGVIVNPKGEMKGSAITG
AtRPL23aA           LDHY-----------QILKYPLTTESAMKKIEDNNTLVFIVDIRADKKKIKDAVKKMYDI
AtRPL23aB           LDHY-----------QILKYPLTTESAMKKIEDNNTLVFIVDIRADKKKIKDAVKKMYDI
                     *                      :      :*.    .

AtRPS6A             QRLVTPLTLQRKRARIADKKKKIAKANSDAADYQKLLASRLKEQRDRRSESLAKKRSRLS
AtRPS6B             QRLVTPLTLQRKRARIADKKKRIAKANSDAADYQKLLASRLKEQRDRRSESLAKKRSRLS
OsRPL23-1           ------------------------------------------------------------
OsRPL23-2           P----------IGKECADLWPRIASAANAIV-----------------------------
AtRPL23aA           QTKKVNTLIRPDGTKKAYVRLTPDYDALDVANKIGII-----------------------
AtRPL23aB           QTKKVNTLIRPDGTKKAYVRLTPDYDALDVANKIGII-----------------------

AtRPS6A             SAAAKPSVTA
AtRPS6B             SAPAKPVAA-
OsRPL23-1           ----------
OsRPL23-2           ----------
AtRPL23aA           ----------
AtRPL23aB           ----------
```

Figure S4. Alignment of RPS6A/B and RPL23 proteins of *Arabidopsis thaliana* and *Oryzasativa* ssp.*japonica*. The conserved Ser/Thr phosphorylation peptide sites are highlighted in red boxes.

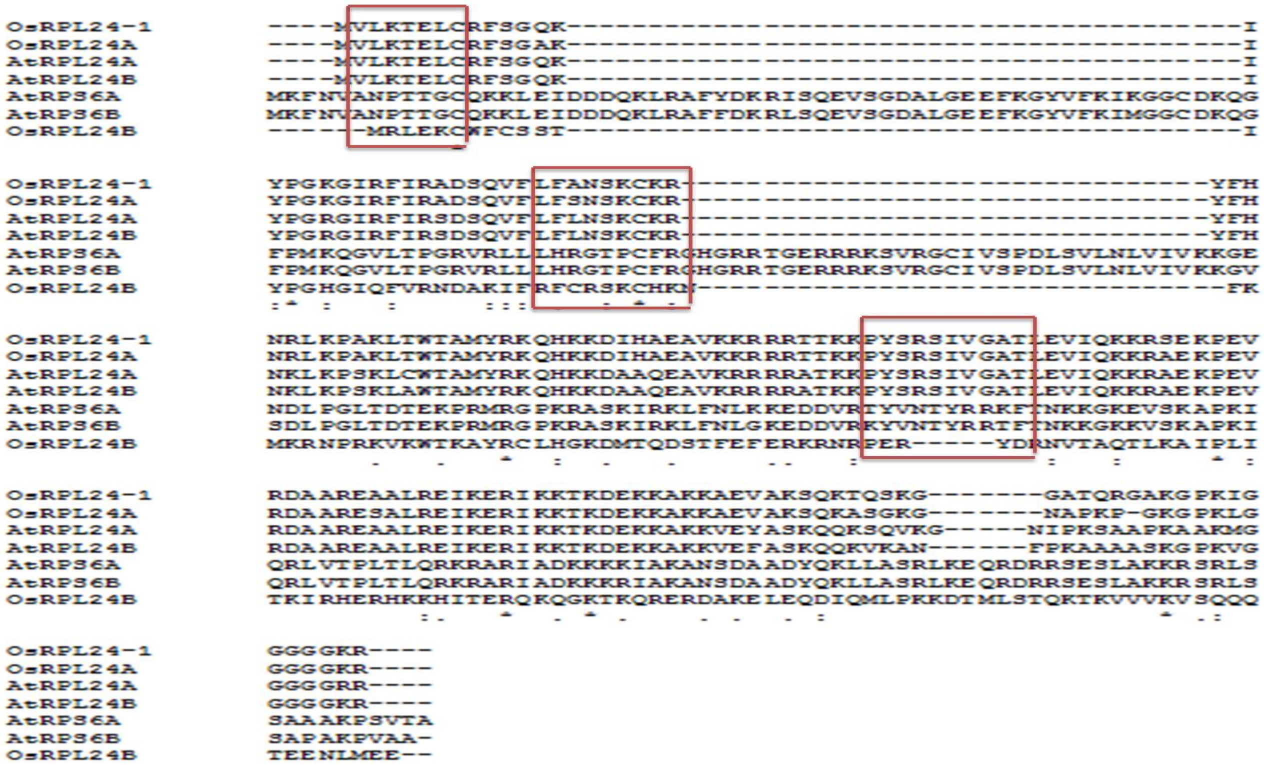

Figure S5. Alignment of RPS6A/B and RPL24 proteins of *Arabidopsis thaliana* and *Oryza sativa* ssp. *japonica*. The conserved Ser/Thr phosphorylation peptide sites are highlighted in red boxes. The Thr81 of AtRPS6 (LHRGT) is replaced by Ser in AtRPL24 and OsRPL24 with peptide sequences LFLNS and LFANS respectively. The above results suggested that the RSKs possibly phosphorylate and activate other RPs in the same manner as they phosphorylate RPS6 protein.

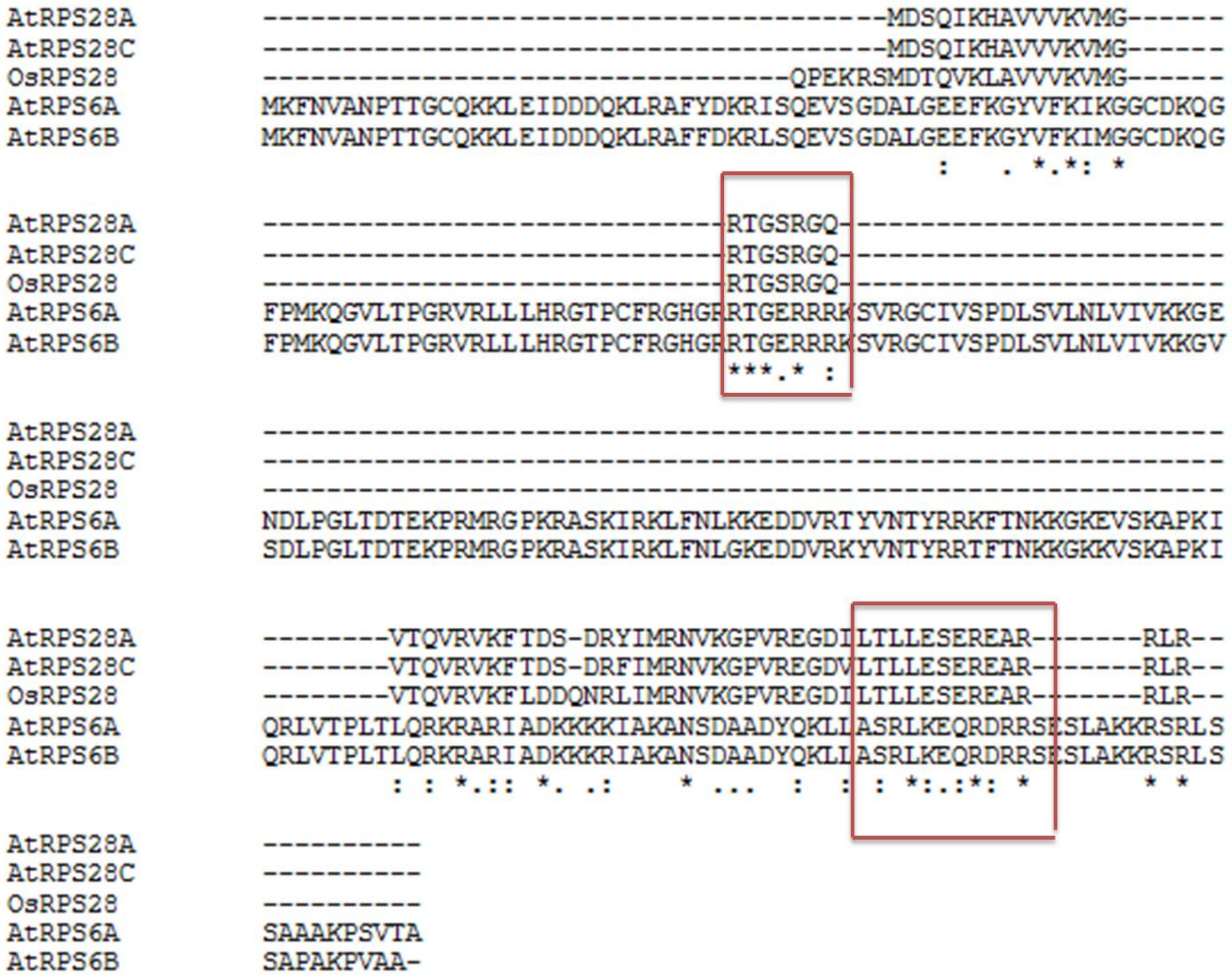

Figure S6 Alignment of RPS6A/B and RPS28proteins of *Arabidopsis thaliana* and *Oryzasativa* ssp.*japonica*. The conserved Ser/Thr phosphorylation peptide sites are highlighted in red boxes.

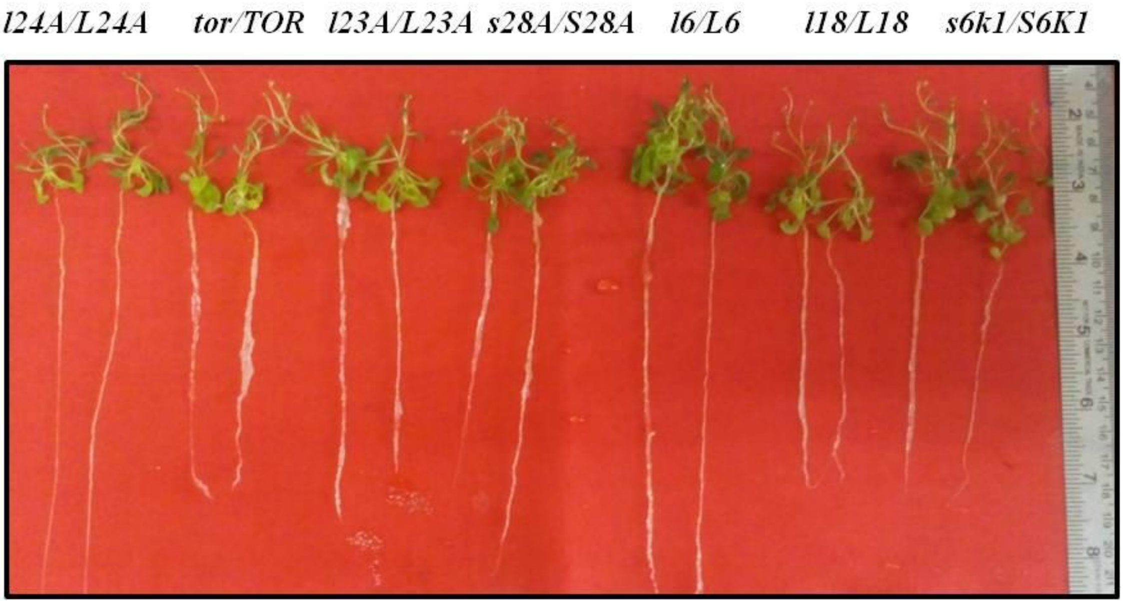

Figure S7. Phenotype of heterozygous T-DNA Arabidopsis mutants of ribosomal protein genes in T_1 generation

The T-DNA instertional mutants of RP genes in T_1 generation were grown on ½ strength MS medium supplemented with 3% sucrose showed pre-mature flowering and differential root-shoot growth. From left to right; SALK lines *rpl24a* (SALK_064513), *tor* (SALK_138622), *rpl23A* (SALK_091329), *rps28A* (SALK_094189), *rpl6* (CS16176), *rpl18* (SALK_134424C), *Ats6k1* (SALK_113295).

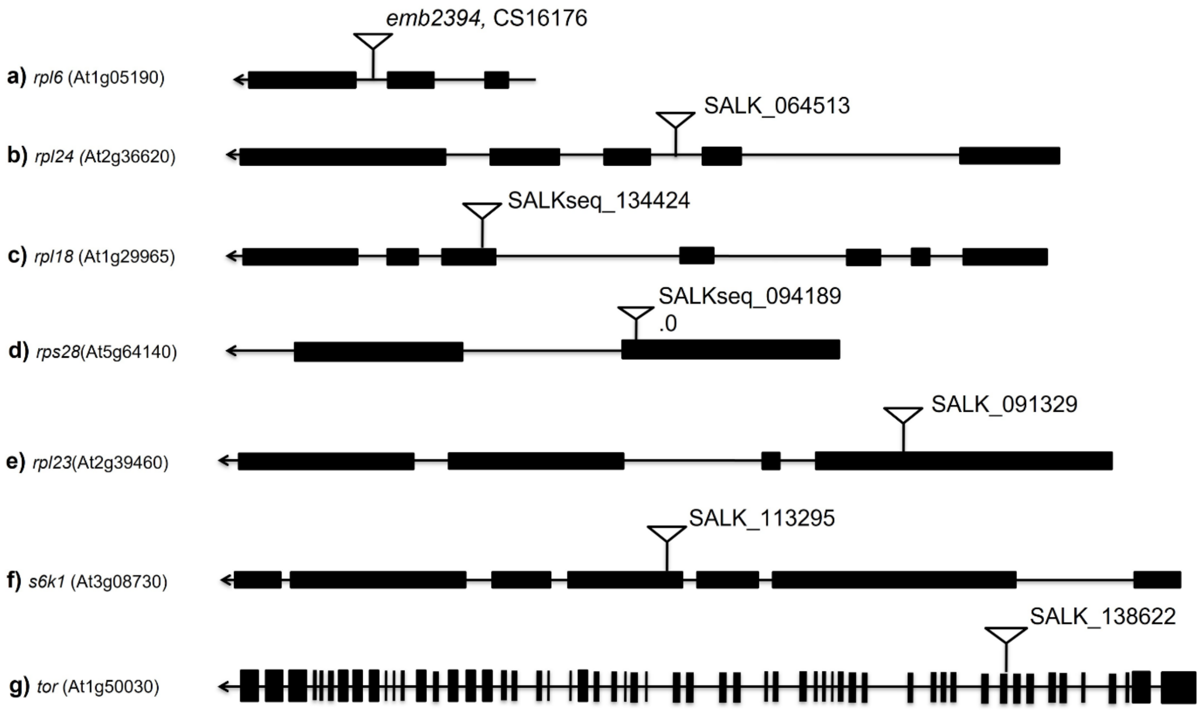

Figure S8. Positions of T-DNA insertion site in the Salk Arabidopsis mutant lines

Maps representing the location of T-DNA insertions in the (a) *rpl6* (CS16176), (b) *rpl24* (SALK_064513), (c) *rpl18* (SALK_134424C), (d) *rps28* (SALK_094189), (e) *rpl23* (SALK_091329), (f) *s6k1* (SALK_113295.1), and (g) *tor* (SALK_138622) mutants of Arabidopsis.

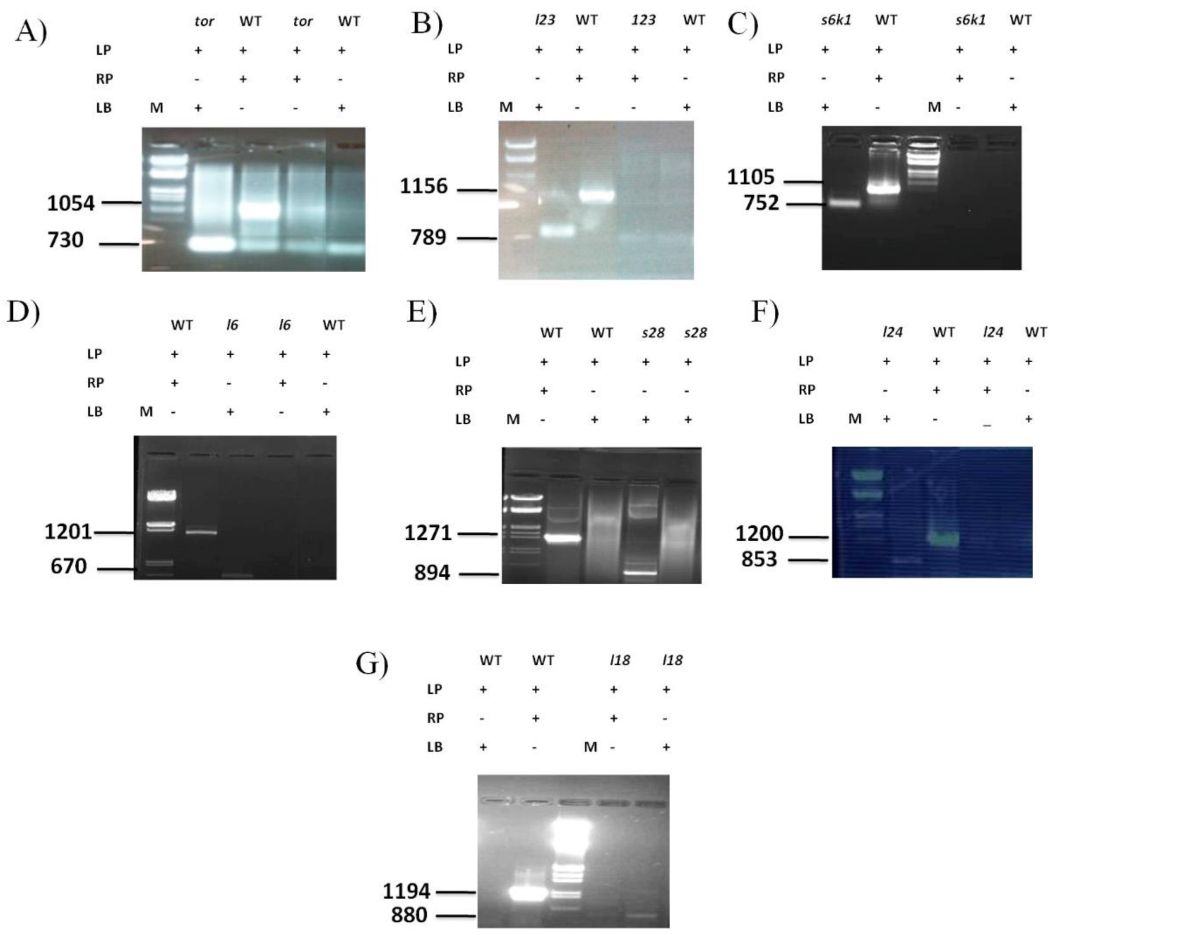

Figure S9. Genotyping of mutants for confirmation of T-DNA insertion

Genotyping of T-DNA insertion alleles of (a) *tor/tor* (b) *rpl23/rpl23* (c) *s6k1/ s6k1* (d) *rpl6*/*rpl6* (e) *rps28/rps28* (f) *rpl24/rpl24* (g) *rpl18/rpl18* homozygous mutants of Arabidopsis. Mutant genotypes were verified by performing genomic PCRs using gene specific Left primers (LP), Right primers (RP) and T-DNA left border specific primer. The homozygous nature of mutants was confirmed by conducting PCR on genomic DNA using the gene specific primers LP and RP to amplify the gene in which T-DNA is inserted. The primers for genotyping of mutants were designed by using T-DNA Primer design website, T-DNA Express database http://signal.salk.edu/cgi-bin/tdnaexpress and protocol for genotyping of mutants was obtained from http://signal.salk.edu/tdnaprimers.2.html. The three universal primers LBa1, LB6313R and LBb1 of T-DNA left border and gene specific LP and RP were designed. Genomic DNA was isolated from the insertion mutant lines using CTAB extraction method and 100 ng genomic DNA template was used for PCR reactions to produce a single amplicon following 35 cycles including 3 min at 94°C, 45 s at specific annealing temperature ranging from 50°C-60°C followed by 2 min at 72°C using either LP + RP primers for amplification of the mutant genes in the WT or heterozygous lines. The LB + RP primers for the forward orientation T-DNA insertion mutants

were used to amplify the length between mutant gene and the T-DNA left border in the homozygous and heterozygous lines. The primer sequences, amplicon length and details of the PCR conditions used in genotyping of mutants are listed in the Supplementary Table S4. The LP and RP amplifies gene in the WT and hemizygous or null mutants (Supplementary Table S4). The primers specific to the left border of T-DNA insert (LB) and RP were used to amplify the products of left border and 3’ end of the gene, which was amplified only in the plants hemizygous and homozygous in nature. For each mutant line a minimum of 10 plants were screened for homozygosity.

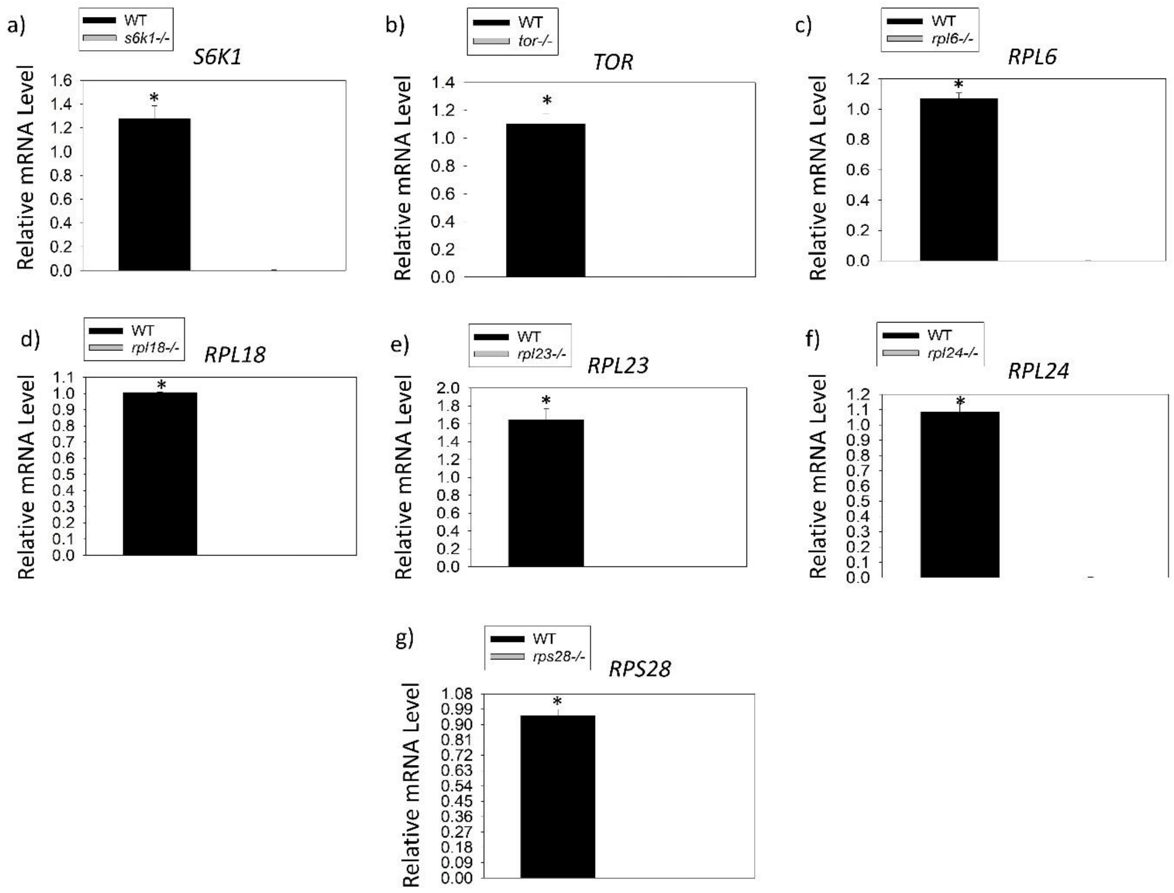

Figure S10. Measurement of transcript level of the mutant allele in the SALK lines

The Relative mRNA accumulation of the T-DNA inserted allele was analyzed in the SALK lines with homozygous alleles with T-DNA insertion of a) *s6k1-/-* b) *tor-/-* c) *rpl6-/-* d) *rpl18-/-* e) *rpl23-/-* f) *rpl24-/-* and g) *rps28-/-* genes using qRT-PCR. The data was normalized using *Act*2 and α*tub* as endogenous reference genes and WT as control. Vertical bars indicate the mean ± SE of three independent biological and three technical experiments and ANOVA analysis indicated the statistically significant differences, represented with asterisks (*) P<0.05.

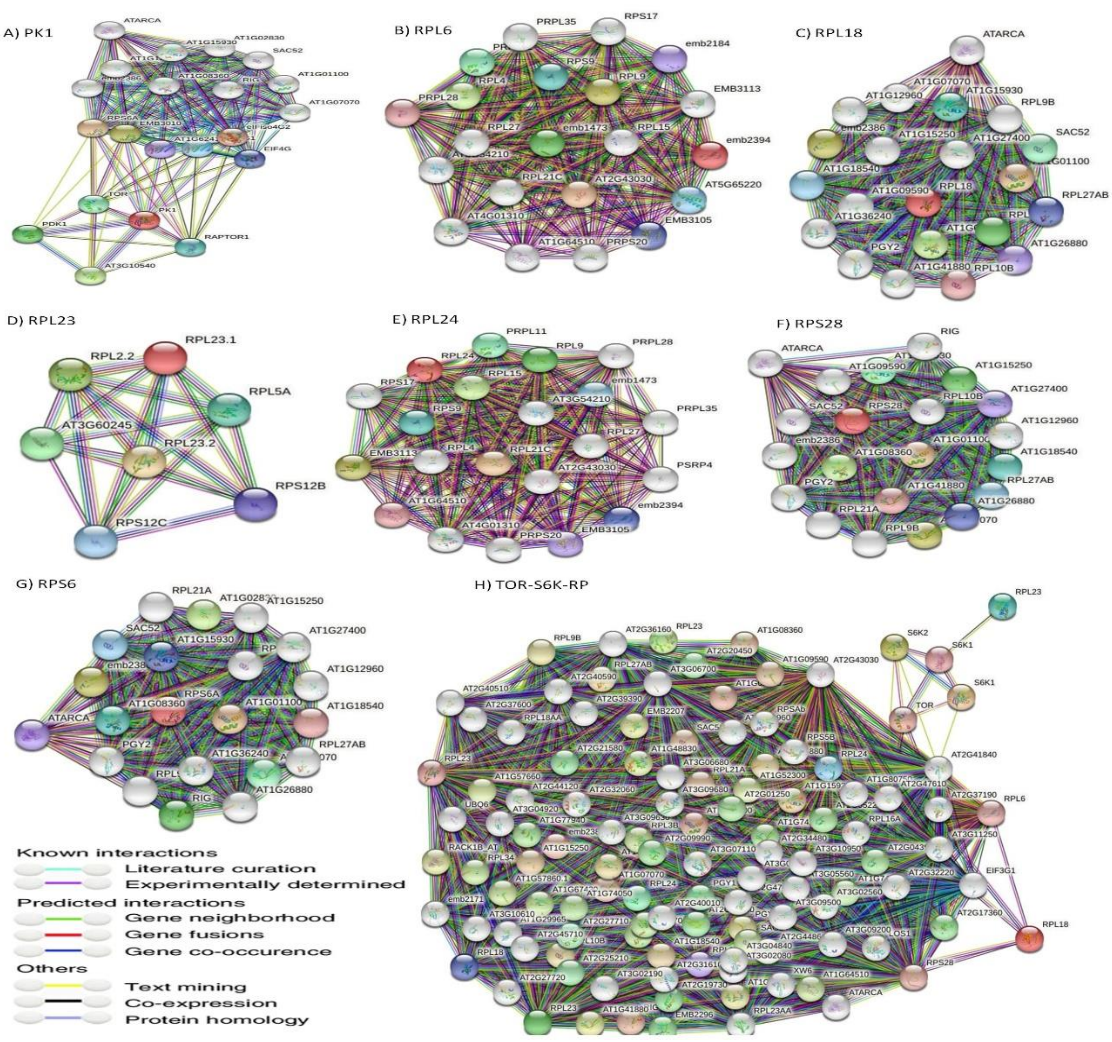

Figure S11. Identification of interacting proteins of TOR-S6K-RPs signaling.

Schematic representation of the PPI network consisting of interactions between input proteins was obtained based on evidence present on high-throughput lab experiments, curated databases, and gene expression data of their direct interactions. The PPI networks of (A) S6K1 (PK1), (B) RPL6, (C) RPL18, (D) RPL23, (E) RPL24, (F) RPS28, (G) RPS6, and (H) the TOR-RPS6K1-RP networks were analyzed separately. The red color node indicates the query protein, and the different colors of nodes represent different interacting proteins relative to the query protein. Different colors of lines connecting the proteins within the PPI network show various modes of interaction. The RPL24 (AT2G36620) protein interacts with the other structural constituents of ribosome assembly such as RPL19e (emb2386, AT1G02780.1), RPS26e (AT2G40590), RPS5 (AT2G41840), RPL16A (AT2G42740.1), RPL18e/L15 family protein (AT2G47570), RPL10 or RPP0 (AT3G09200), RPL4/L1 (AT3G09630), RPS12/S23 (AT3G09680), RPS17 (AT3G10610), RPS24e family (AT3G04920), RPS28

(AT5G64140.1), RPL23AA (AT2G39460.2), RPL11 (AT2G37190), RPS5B (AT2G37270.2), RPS11 (AT2G36160), Zinc-binding RPL37ae (AT3G10950), PGY2 (encodes RPL9, AT1G33140.1), RPL35Ae (AT1G41880), Translation protein SH3-like family protein RPL21e (AT1G57660), RPL3B (AT1G61580.1), Senescence associated gene 24 (SAG24, AT1G66580.1) and RPL22p/L17e (AT1G67430). RPS28C (AT5G64140) interacts with ribosomal constituents RPL23 (ATCG00840.1 & ATCG01300.1), RPL21A (AT1G09590.1), RPL18 (AT3G05590.1), RPL16A (AT2G42740.1), RPL10B (AT1G26910.1), RIG (AT1G04270.1, encodes a cytosolic RPS15), RACK1B_AT, PGY2 (AT1G33140.1, encodes RPL9), PGY1 (AT2G27530.1; encodes RPL10aP),

Table S1. List of primers used in RT-qPCR analysis of RPS genes in *TOR*-OE lines of rice

S. No.	Primer name	Sequence 5'- 3'
1.	S3a RT FP<br>S3a RT RP	TGAAGCCCATGTGGATGTTA<br>TCACATGTTGATGCCTGGTT
2.	S4 RT FP<br>S4 RT RP	CAGGTTGAAGTATGCGCTGA<br>AGCGACCCTTGGTGTCATAG
3.	S4a RT FP<br>S4a RT RP	AGCGGCATGTTATGGTTGAT<br>GGACCTTGCAGAGCTTGAAC
4.	S5 RT FP<br>S5 RT RP	ATATCCCTCGCCGACTACCT<br>TGATCTTCTTGCCGTTGTTG
5.	S5a RT FP<br>S5a RT RP	CAACGGGAAGAAGATCATGG<br>GACCCTCCTCAAGGGAGAGA
6.	S6 RT FP<br>S6 RT RP	GCTACGGCATCAAGAAGCTC<br>CAAGTGCCGTGTCACAAAGT
7.	S6a RT FP<br>S6a RT RP	CATGAAGCAGGGTGTGCTTA<br>TCAGTCAAGCCAGGAAGGTC
8.	S7 RT FP<br>S7 RT RP	TGTGCAAGGCATTCAAGAAA<br>TCCAAGATTCCATCATGAACAG
9.	S7a RT FP<br>S7a RT RP	AGAAGAAGTTCAGCGGCAAG<br>GCACCATCCAGACGGTATCT
10.	S9 RT FP<br>S9 RT RP	TTATCAGGCAGCACCACATC<br>GGCCTTCTGGTTCTTCCTCT
11.	S9-2 RT FP<br>S9-2 RT RP	GAGCTGTGGCGTGTTCAGTA<br>AACAGTGAGGGCAAGGACAT
12.	S10 RT FP<br>S10 RT RP	CCCTCAAGAAGTCTGCCAAG<br>ACCAAAATCACCTGGAGCAC
13.	S10a RT FP<br>S10a RT RP	GCATTGAGCACCTGAGGAAT<br>AACCTTGGTCTGTCCCCTTC
14.	S13 RT FP<br>S13 RT RP	AGGTGGAGGAGATGATCGTG<br>GACGGCCTTCTTGATGAGG

15.	S13a RT FP<br>S13a RT RP	GTGGTGCTCCGTGACCAG<br>TGGAGTCCTTGTCCTTCCTG
16.	S15 RT FP<br>S15 RT RP	ATGACCTCGTCCAGCTCTTC<br>CCGATCATCTCAGGGACAAT
17.	S15a RT FP<br>S15a RT RP	CCGACCCTCATCAAAGGTTA<br>TCCTTGACACCAACATCGAA
18.	S17 RT FP<br>S17 RT RP	AACAAGAAGGTGCTGGAGGA<br>TCCTGGAGCTTGAGGGAGAT
19.	S18 RT FP<br>S18 RT RP	CGACATCGACATGAACAAGC<br>TCAGCCTCTCAAGGTCATCC
20.	S18a RT FP<br>S18a RT RP	CTCACCTCCATCAAGGGTGT<br>ACCTCCCGTCCTTGTAGTCC
21.	S18b RT FP<br>S18b RT RP	AAGGATGGGAGGTTCTCTCAG<br>TCTTGGAGACACCGACAGTCT
22.	S19 RT FP<br>S19 RT RP	CAAGATGGAGCTCCCTGAGT<br>GGACGTGAGCCATTCCTCT
23.	S20 RT FP<br>S20 RT RP	GCACCAGGATGAAGAAGGTT<br>TGCGATGGTTACCAGTATTCC
24.	S21 RT FP<br>S21 RT RP	TCAGATGGTGGACCTCTACG<br>AGAGCACTGTCGGCGTCT
25.	S23 RT FP<br>S23 RT RP	GAGCCATCTTGGCAATGAAT<br>CGATGAAGTTCAAGCAACCA
26.	S23a RT FP<br>S23a RT RP	TTTTGGGAGAAAAAGGCTTG<br>CCTCTGCTTCTTCTTCTCCTTG
27.	S24 RT FP<br>S24 RT RP	GTCTCCAAGGCTGAGCTGAA<br>CTTGGGCTCGTACTTCTTCG
28.	S25 RT FP<br>S25 RT RP	GGGAAAGCAAAAGGAGAAGG<br>GATTAGGCCCCGTGTCATTA
29.	S25a RT FP<br>S25a RT RP	CGGAGGCAAGCAGAAGAAG<br>CAAGATCCTTGATGGCCTGT
30.	S26 RT FP<br>S26 RT RP	CAAGGCGATCAAGAGGTTTC<br>GACGATGTGAGCATGGATTG
31.	S27 RT FP<br>S27 RT RP	CGGAGCTGGAGAAGCTCA<br>CTGGCCTTCCCACCAGTAG
32.	S27a RT FP<br>S27a RT RP	CGAAGATCCAGGACAAGGAG<br>CTTGGGCTTGGTGTACGTCT
33.	S28 RT FP<br>S28 RT RP	GGATACCCAGGTCAAACTTGC<br>CTGGCCTCCCTCTCAGACT
34.	S29 RT FP<br>S29 RT RP	CACTCCAACGTGTGGAACTC<br>CGGTACTTGATGAAGCCAATG
35.	S30 RT FP<br>S30 RT RP	GAAGGTGAGGGGGCAGAC<br>GACGAAACGGCGGTTGTACT
36.	OsActin1RT FP<br>OsActin1RT RP	TCCCCCATGCTATCCTTCG<br>TGAATGAGTAACCACGCTC
37.	OsTubulinRTFP<br>OsTubulinRTRP	TGACCACACCTAGCTTTGG<br>AGGGAACCTTAGGCAGCATG

Table. S2. List of primers used in RT-qPCR analysis of RPL genes in *TOR*-OE lines of rice

S. No.	Primer Name	Sequence (5'-3')
1.	RPL3 RT FP<br>RPL3 RT RP	TGGACTTGTGGCCTATGTGA<br>CCGGCATCGCTATCATACTT
2.	RPL4 RT FP<br>RPL4 RT RP	AAGAAGCTCGACGAGGTGTA<br>CCACATTCTTCAGAGGGTTC
3.	RPL5 RT FP<br>RPL5 RT RP	GATCTTGGCATCAAGTACGAC<br>GACACCCTCATACTTGACCTG
4.	RPL6 RT FP<br>RPL6 RT RP	GTTCCTCAAGCAGCTCAAAT<br>CTTCTGCTTCTTGTCCCTAGA
5.	RPL7 RT FP<br>RPL7 RT RP	TACCCAAACCTGAAGAGTGTC<br>GACAGTCATGATCTCGTGGA
6.	RPL8 RT FP<br>RPL8 RT RP	ACTACGCCATCGTCATCAG<br>GGTACTTGTGGTAGGCGTTT
7.	RPL10 RT FP<br>RPL10 RT RP	AGAAGAAGCCTGGATTAGAGC<br>ATATCCTGCTGGAGGACTTG
8.	RPL11 RT FP<br>RPL11 RT RP	AAGAAGATCGGTGAGGACATC<br>TCTTGACCTTCTTCCTGTCC
9.	RPL12 RT FP<br>RPL12 RT RP	GCTCATTTGTACAGCACAGAG<br>TTGGTTCAGTCTGAGAAGGAG
10.	RPL13a RT FP<br>RPL13a RT RP	GAACTACCACGACACCATCAG<br>GGGGCCAAAATATCTATCTG
11.	RPL13b RT FP<br>RPL13b RT RP	AAGCACTGGCAGAACTATGTC<br>CCCTCGACTTCATGTTGTACT
12.	RPL14 RT FP<br>RPL14 RT RP	GTGAACTACGGCAAGGACTAC<br>TAACATCAGCCTCCTCCATAG
13.	RPL15 RT FP<br>RPL15 RT RP	ACAAGTACGTGTCGGAGCTAT<br>GACACGGTAAACCACATAACC
14.	RPL18a RT FP<br>RPL18a RT RP	TCCAAGTTCTGGTACTTCCTG<br>GTTGTGGTAACCTGTTCTGCT
15.	RPL18p RT FP<br>RPL18p RT RP	TGGGGAGGACTACTATGTTGA<br>AAACCTCTTGTCACTGTGAGG

16.	RPL19.3 RT FP<br>RPL19.3 RT RP	AGTATCGTGAGGCCAAGAAG<br>CTTAGCCTCAAACTGGTCAGA
17.	RPL21.2 RT FP<br>RPL21.2 RT RP	CTGAGGAAGATCAAGAACGAC<br>AACCACCCTTGAGATCATTG
18.	RPL22 RT FP<br>RPL22 RT RP	GAGGTGAAAGGTCTGGATGTT<br>TCACTGGTTCTTCCTTCTCTG
19.	RPL23A RT FP<br>RPL23A RT RP	GACCAAAGACCCTGAAGAAGG<br>ACGATGAAGACAAGGGTGTTG
20.	RPL24b RT FP<br>RPL24b RT RP	GTTGGTGCTACACTGGAAGTT<br>CCTTCGACTGTGTCTTCTGAG
21.	RPL26.1 RT FP<br>RPL26.1 RT RP	ACAAGTACAACGTGGTGAGG<br>GTCCTTGTCGAGCTTGAGTT
22.	RPL27.3 RT FP<br>RPL27.3 RT RP	CTTCCTCAAGCTCGTCAACT<br>CTTGGTGAAGAACCACCTGT
23.	RPL28 RT FP<br>RPL28 RT RP	TAGACGAATACCTCCTGAAGA<br>AAACCCTGTTCGATCTTAGTC
24.	RPL29 RT FP<br>RPL29 RT RP	CCCAACAAGCTCTCCAATATA<br>AGAAACAGAAGCATTCCCTG
25.	RPL30e RT FP<br>RPL30e RT RP	GAGCAAGAAGAAGAACAAGTC<br>GCTTCATCCATATCTTTTCCG
26.	RPL31 RT FP<br>RPL31 RT RP	TCAAGGAGATCAGGAAGTTTG<br>AACAGTGACCAGAGAGTAGAG
27.	RPL32 RT FP<br>RPL32 RT RP	GCCTAATATTGGCTATGGTTC<br>CTTCTTCGTTGAGACATTGTG
28.	RPL34 RT FP<br>RPL34 RT RP	GAAGAAGATCCAGGGAATTCC<br>CACAATCTTCTGCTCTTCAAC
29.	RPL35a.3 RT FP<br>RPL35a.3 RT RP	CTACGTCTACAAGGCCAAG<br>TGCTGGGGTACATGAAGA
30.	RPL36.2 RT FP<br>RPL36.2 RT RP	GGAAAAGTACCAAGAGAGTGA<br>CTTCTTCTTTGCTCTCTTGTG
31.	RPL37 RT FP<br>RPL37 RT RP	CTTCCACCTGCAGAAGAG<br>CCCCTCTCTGAAGTTACTCT
	RPL38 RT FP	CACGAGATCAAGGACTTCC

32.	RPL38 RT RP	AAAGGTGGATGAAATGTAGGC
33.	RPL44 RT FP<br>RPL44 RT RP	AAGAAGACCTACTGCAAGAAC<br>CCTTACCCTTCTTGTACTGAG
34.	RPL51 RT FP<br>RPL51 RT RP	GTGACAGAGTTAGTCCGTGGA<br>TCTCAGCTTCACCACTTTCCT

Table. S3. List of primers used in RT-qPCR analysis of RPL/RPS genes in the *TOR*-OE lines of *Arabidopsis thaliana*

S. No.	Accession	Primer name	Sequence (5’-3’)
1.	At1g72370	RPSaAFP<br>RPSaARP	CCTGGTACCTTCACCAATCAGATGC<br>GTTGTTGGCTGGGATACCAATGTCA
2.	At1g58380	RPS2FP<br>RPS2RP	AAGGCCTTTGTTGTTGTTGG<br>CCCAATCTTATTCCCCCAGT
3.	At2g31610	RPS3AFP<br>RPS3ARP	TGGAGCTAAGGGATGTGAGGTCATC<br>AATGGTGTCTTTGGTCCTGATTTGC
4.	At3g04840	RPS3aFP<br>RPS3aRP	GCAACTCAGGGCATCTACCCTC<br>CAGCCGGCCTGTCTACCTTAACA
5.	At2g17360	RPS4FP<br>RPS4RP	TGCTGGTTTCATGGATGTTG<br>CATGGCTTTGTTCCCTTACCG
6.	At2g37270	RPS5aFP<br>RPS5aRP	GCCGCGGCTAAAGATCCCTACTT<br>GGCACTGCGCCTTTCTGAACCT
7.	At4g31700	RPS6aFP<br>RPS6aRP	CGATCTTCCTGGGCTTACCGAT<br>CCTCTGGATCTTAGGGGCTTTG
8.	At5g10360	RPS6BFP<br>RPS6BRP	GCCGCGTCGAGAGCATTTTAT<br>GAAACCTTGCTTGTCACATCCT
9.	At3g02560	RPS7BFP<br>RPS7BRP	TGGAACACCGAAGCTCTGGATTAAC<br>GGTGCAACACCCTTATCCTTGTGG
10.	At5g20290	RPS8AFP<br>RPS8ARP	TCGGCGACAATGGGTATTTCTCG<br>CAACGCACGCCACTTAACATTTCC
11.	At4g12160	RPS9 FP<br>RPS9RP	GGAGCGTCGTCTTCAGACTATT<br>GTGCTAGCTTCCAAGAGTGTTCTC
12.	At4g25740	RPS10FP<br>RPS10RP	AAGCTCAACCAACAAGACG<br>AGCAGGGACAACATCGGAAGG
13.	At3g48930	RPS11FP<br>RPS11RP	TACTTGCCACAGTGCGAAAATGC<br>GCCGGAATGTTTGAATGCCTCT
14.	At1g15930	RPS12AFP<br>RPS12ARP	TGCTGATCACGAAGTCAGGTTGC<br>CTCGCCGAAGTCCTTGACAACA
15.	At3g60770	RPS13 FP<br>RPS13 RP	AGCAGCAGCCGTAAGAAAGAA<br>AGCAGATGCCGAGATACCC
16.	At2g36160	RPS14A FP<br>RPS14ARP	CCTGGAGCACAGTCTGCCCTTAGA<br>CCACAATGCGAGAAAAACTCAGAGC
17.	At1g04270	RPS15A FP<br>RPS15ARP	CCTCTCAGCAGCCAACGACAGTTAT<br>CCCTTTTCGCTTTCCTCAGCTTC

18.	At1g07770	RPS15aA FP<br>RPS15aARP	GCCCACGTTTTGATGTTGGTGTC<br>CCAGCAGATGTAGTCAGCACGATG
19.	At5g18380	RPS16C FP<br>RPS16CRP	TACGCCATCCGTCAGAGTATC<br>AGCAGAGTCCTATCATACCTCACC
20.	At2g05220	RPS17BFP<br>RPS17BRP	ATCTCTGTCTCCGGTAAGTCACTG<br>ACTGGTCCCTTCTGGATACGTT
21.	At1g22780	RPS18 FP<br>RPS18 RP	GTTGCTAACCCTCGCCAGTTCA<br>ACACGGAGACCCCAGTAGTGC
22.	At3g02080	RPS19 FP<br>RPS19 RP	TGGAAGCAAGAGGAACGGTA<br>TCCCTTTGGCCACTGGAAGTGA
23.	At3g47370	RPS20 FP<br>RPS20 RP	CTCCGCAGTTGCACACAAATT<br>TGGGCTTCATCGGTTCATACGC
24.	At3g09680	RPS23 FP<br>RPS23 RP	ACGTCGCGCCTTTAGCAGTTTC<br>TCCGCCCACCTCTGAGTAATCC
25.	At5g28060	RPS24 FP<br>RPS24 RP	TGGCGGAAAAAGCTGTCACT<br>TTTGAAACATTGGCTCTTC
26.	At2g16360	RPS25AFP<br>RPS25ARP	CGGTGGCGGAAAGCAGAAAAAG<br>CAAGCGACCCATTGATCCGAAG
27.	At2g40510	RPS26 FP<br>RPS26 RP	CGGCAACAAGAGCGTAAACCC<br>TCGGCAATGTGTATCCCTCAT
28.	At2g45710	RPS27A FP<br>RPS27A RP	CGGTTGATTCCATACGATCTCCAAG<br>CGAATTGGGTGATTGAACAAGACG
29.	At1g23410	RPS27BFP<br>RPS27B RP	GGTTCTACGTCTGAGAGGAG<br>CCACAACTAACAGAAGGACAC
30.	At3g10090	RPS28 FP<br>RPS28 RP	TCTACCCATCGCAGAGCCTC<br>CTCCAGCAAGGTGAGAATATC
31.	At3g43980	RPS29AFP<br>RPS29ARP	GGGTCACTCTAATGTCTGGAACTC<br>GTTACTGCGGAAACACTGTCTG
32.	At2g19750	RPS30 FP<br>RPS30 RP	GGTGAGAGGTCAGACACCT<br>GAGTTTGGTCCTCTCTTCTTGC
33.	At1g43170	RPL3 FP<br>RPL3 RP	GAAGGCGTTCCCTAAGGATG<br>GTGCCCAGACAGTGTTCAAAG
34.	At3g09630	RPL4AFP<br>RPL4ARP	GAGGAAAACCGTTACCAAGGAGGAG<br>CTGCTGGGGTAAACCAAAGAAAGC
35.	At3g25520	RPL5AFP<br>RPL5ARP	TGTTCGGTGCTCTTAAGGGTGCTT<br>TGGGTTGGGGTCAGCACGAATAG
36.	At1g18540	RPL6 FP<br>RPL6 RP	GGCGAAGAGGACTCCCAAG<br>AGTGATGCTGGCTTTTAGCTT
37.	At1g80750	RPL7FP<br>RPL7RP	GGATGCACAATCATTGAGGGAAATC<br>TCTTCCGGTGCAAAACATCAGC
38.	At2g47610	RPL7a FP<br>RPL7a RP	GGTCCCTCCAGCTCTTAACCAA<br>CACATGGTTGAGGCCGTATTT
39.	At2g18020	RPL8FP<br>RPL8RP	CGGCGAGAGAAATGGTTACCTCA<br>TCGAACCCGATGGCAACTTAATC
40.	At1g33120	RPL9 FP<br>RPL9 RP	TGTTGGATGGTGTAACCATTG<br>GTGGATTTGGTCCATAAAAC
41.	At1g08360	RPL10a FP<br>RPL10a RP	CCACATATCCCCCGTCCTAA<br>CCATGCACAGAACCTTCTTCAG
42.	At2g42740	RPL11AFP	GATCGCATGCTATGTAACCGTCAGA

		RPL11ARP	TAGCCAGGACGTTCTAGGACCACA
43.	At2g37190	RPL12AFP<br>RPL12AFP	CGGAGAGAGATAGGAAGAAGGTG<br>CCACAGTACAACCAACAGACACAC
44.	At3g48130	RPL13FP<br>RPL13RP	GGCGTATAACACTCTTCGTGTAGC<br>GACGCTCACTTCTTCTCTTCGT
45.	At3g07110	RPL13a FP<br>RPL13a RP	TGGGTCGCCTTTCTTCTGAAGTCG<br>GGAGGCGCATGACAAAAGACG
46.	At2g20450	RPL14FP<br>RPL14RP	TACGGCGAGGATTATGGAAAGCTC<br>GGCCAACATGATCTTGAACCTGTCA
47.	At4g17390	RPL15FP<br>RPL15RP	CGTGTTCGAGTCAGACGTGGTG<br>CCTGCGGGAAGGTCTGTTCTTG
48.	At1g27400	RPL17aFP<br>RPL17aRP	TCAGGCAAAGAACAGGCATTCG<br>GATGTGAGAAATGAAAAGGGCATCG
49.	At2g47570	RPL18FP<br>RPL18RP	CTCTCTCGTCTGGTCCGATACATGG<br>GCGCTCTCAGTGAACCTCAATGC
50.	At1g29970	RPL18a FP<br>RPL18a RP	GCAACCACAGCATCCGAT<br>CGCGGTGATCAACAGATGTGAC
51.	At3g16780	RPL19FP<br>RPL19RP	AGGGTCGTCACTCTGGATACGGTAA<br>GCCTTCTCAGCCTTCATCTTGTGG
52.	At1g09590	RPL21 FP<br>RPL21 RP	GCGAGAACGAGGGATCTGTTCG<br>CTCCTCGGCACATCTTGACTGC
53.	At5g27770	RPL22FP<br>RPL22RP	CAGGAGAGGATTAAGGTTGGTG<br>CTTCTTCCTCAGCTTCGTTCTC
54.	At1g04480	RPL23FP<br>RPL23RP	CGACGGTGAACTGTGCCGATAA<br>CCTTGGGGTTGACAATGACTCCA
55.	At2g39460	RPL23a FP<br>RPL23a RP	AAGGCTGTGAAGTCTGGTCAAG<br>CCAGTTCTAGGCTTGGTAAGAGTC
56.	At2g36620	RPL24 FP<br>RPL24 RP	TCGATTGTCGGTGCTACTTTGG<br>CCACCACCACCCATCTTAGCAG
57.	At3g49910	RPL26AFP<br>RPL26ARP	GATAATGAGCTCTCCTCTCTCCAC<br>CTTCACGTCCCTTGTAAGTACCAC
58.	At1g12960	RPL27a FP<br>RPL27a RP	CGCCGGTGTTGGTATGAGGTATT<br>CCGGCAAGAAACCTTTCCCTAAA
59.	At2g19730	RPL28FP<br>RPL28RP	GGAATTCCCCAGGATGTCAAAGG<br>GCTGATGGCGCTGAGTCTAGCA
60.	At3g06700	RPL29FP<br>RPL29RP	CGAGCAGATCTACCTCTCTCTCTC<br>GGAACTTAGGGTCCATTCCTCT
61.	At1g77940	RPL30BFP<br>RPL30BRP	CCACCGCACAGATCCAAGACAT<br>CGCAAGCTGTTCCCAAATCAACG
62.	At2g19740	RPL31aFP<br>RPL31aRP	CTCCAACACCATTTCGAAGACCAG<br>CGGGCAACACGGACTCTAATTCTC
63.	At4g18100	RPL32 FP<br>RPL32 RP	GCGGTCCCATTGCTTACCAAGAA<br>AACATTGGGCATCAAAGTCACAC
64.	At1g26880	RPL34 FP<br>RPL34 RP	TGGCCCCAAATGTCCTGTTACTG<br>CCCGAATGATCCTTTCCCTGACTG
65.	At3g09500	RPL35FP<br>RPL35RP	GCTCTCCTCCGTGTCGCTAAAGTC<br>TGGTGAGGCGTCTACGAATAGCA
66.	At1g06980	RPL35aFP<br>RPL35aRP	CCTCGTCTTCTCCAACTTGTTC<br>CTTAGGCTGTTTCCCATCTCTCTC

67.	At2g37600	RPL36FP<br>RPL36RP	TCGTTGGATTGAACAAAGGACACG<br>CACGCTTGTCTTTCCCAACCTTGA
68.	At3g23390	RPL36a FP<br>RPL36a RP	AGATCAGAGACGCGAAAATGGT<br>TCACCACCGATCTCGAAATGCTT
69.	At1g15250	RPL37FP<br>RPL37RP	GCGGCAATGGGTAAAGGAACAG<br>TGAACCTTCGAGGGACATTACGG
70.	At2g43460	RPL38AFP<br>RPL38ARP	GGCGGCTAGTTTCAACCAACAGA<br>TGCAAAGGATTCGTCACCAAAGGA
71.	At2g25210	RPL39 FP<br>RPL39 RP	GGCCCAGAACAAGATCGCAAGA<br>CCTGATGGTGTTGTCGGTACGAA
72.	At2g36170	RPL40 FP<br>RPL40 RP	TTGCATCTTGTTCTGAGGCTTA<br>TGGCCTCAACTGGTTGCTATGG
73.	At3g08520	RPL41 FP<br>RPL41RP	ACAGCTTCTGCGAATCAGC<br>CGAATCTACTTAGATCGCTGTC
74.	At3g18780	AtActin2 FP<br>AtActin2 RP	CAGCAGATGTGGATCTCCAAGG<br>CGCAGACGTAAGTAAAAACCC

Table. S4. List of primers used in genotyping of T-DNA mutants

S. No.	T-DNA lines	Primer details
1.	SALK_138622 (TOR)	LP 5'AACCCTTACATGACATGCTCG 3' Len 21 TM 60.01 GC 47.62, PRODUCT_SIZE 1054<br>RP 5' AATCACCTGCATAACACGCTC 3' Len 21 TM 60.15 GC 47.62<br>Insertion chr1 18524835 BP+RP_PRODUCT_SIZE 430-730
2.	SALK_091329 (RPL23)	LP 5' GCAAAGTTGCTGGAATTGAAG 3' Len 21 TM 59.87 GC 42.86, PRODUCT_SIZE 1156<br>RP 5' ACCCACTCACCACTGAATCTG 3' Len 21 TM 60.02 GC 52.38<br>Insertion chr2 16475960 BP+RP_PRODUCT_SIZE 489-789
3.	SALK_113295 (S6K1)	LP 5' TCTGTATCGATTTTTCCCGTG 3' Len 21 TM 59.95 GC 42.86, PRODUCT_SIZE 1105<br>RP 5' TTTCAGGTGCCATATACTCCG 3' Len 21 TM 59.97 GC 47.62<br>Insertion chr3 2652628 BP+RP_PRODUCT_SIZE 452-752
4.	SALK_134424 C (RPL18)	LP 5' GTGGGAGGTCTAACCTTCCTG 3' Len 21 TM 59.98 GC 57.14, PRODUCT_SIZE 1194<br>RP 5' CACAAAAAGTTCCCCAAAAGC 3' Len 21 TM 60.84 GC 42.86<br>Insertion chr1 10499225 BP+RP_PRODUCT_SIZE 588-888
5.	SALK_064513 (RPL24)	LP 5' AAAACATTTCTTTCATGGGCC 3' Len 21 TM 60.18 GC 38.10, PRODUCT_SIZE 1200<br>RP 5' GAAAGAAACAAACCGTAGGGC 3' Len 21 TM 59.99 GC 47.62<br>Insertion chr2 15351229 BP+RP_PRODUCT_SIZE 553-853
6.	Emb2394, CS16176 (RPL6)	LP 5' ATTCCGCTAGGGTTTCGTTT 3' Len 20 TM 59.69 GC 55, PRODUCT_SIZE 1201<br>RP 5' TTGAGCAAAAGGTGTGCTTG 3' Len 21 TM 59.28 GC 47.62<br>Insertion chr1 1503483 BP+RP_PRODUCT_SIZE 370-670

7.	SALKseq_094 189 (RPS28)	LP 5’ GAATCAATCGGTTCGTGACTG 3’ Len 21 TM 60.51 GC 47.62, PRODUCT_SIZE 1271<br>RP 5’ TACCAACCAGAGCTCAAATTG 3’ Len 21 TM 57.89 GC 42.86<br>Insertion chr5 25668245 BP+RP_PRODUCT_SIZE 594-894
8.	LBb1.3	5’ ATTTTGCCGATTTCGGAAC 3’
9.	LBa1 of pBIN-pROK2 for SALK lines	5’ TGGTTCACGTAGTGGGCCATCG 3’
10.	LB_6313R for SALK lines	5’ TCAAACAGGATTTTCGCCTGCT 3’

Table S5. List of primers used in RT-qPCR of the T-DNA insertion in the SALK lines

S. No.	Gene	Accession	Primer Sequence (5’-3’)
1.	*TOR*FP (Kinase domain)<br>*TOR*RP (Kinase domain)	At1g50030	TACGACCATACAGGAAAGAGCAT<br>TTCTATCAAGGGCAACTGGTTTA
2.	*S6K*1FP<br>*S6K*1RP	At3g08730	GGAGCGTAGGGATTCTTCTGTAT<br>TCTGCTGTATCTTTCCTTTGCTC
3.	*RPL*6FP<br>*RPL*6RP	At1g05190	TTCTTGTCATCCAATGAAGTCCT<br>CTATCCGGTATCTGCATCTTCAC
4.	*RPL*18FP<br>*RPL*18RP	At1g29965	ATATTTTGTCGTTCGTGTTGCTT<br>TCCACTGCTCCATTCAAAGTAGT
5.	*RPL*23FP<br>*RPL*23RP	At2g39460	TCAATCCAAAGCTTACTGGGTTA<br>AGCGAAGCTCAGTAATGAATCTG
6.	*RPL*24FP<br>*RPL*24RP	At2g36620	AGCCATGGTTCTCAAGTAATCAA<br>TTTCTGATCGGGAAATAAACAGA
7.	*RPS*28FP<br>*RPS*28RP	At5g64140	ATTCCACCATATCCCAATTCTCT<br>TGCGTCATAAAAGATTCATTCCT

Table S6. Identification of the predicted functionally interacting partners of TOR-S6K-RP signaling for ribosome biogenesis

PPI network	Predicted Functionally interacting Partners	Gene	Function
**PK1/S6K1 (AT3G08730)**	AT1G18080.1, ATARCA	Transducin/WD40 repeat-like superfamily protein	Major component of the RACK1 regulatory proteins that play a role in multiple signal transduction pathways
	AT1G02830	Putative 60S ribosomal protein L22-1/L22e	Its function is described as structural constituent of

		family	ribosome
	AT1G15930	Ribosomal protein L7Ae/L30e/S12e/Gadd 45 family protein	Its function is described as structural constituent of ribosome;;
	AT1G12960	Ribosomal protein L18e/L15 superfamily protein	Ribosome assembly
	AT1G02780.1, emb2386	Ribosomal protein L19e family protein	Embryo defective 2386 (emb2386) is structural constituent of ribosome
	AT1G14320.1, SAC52	Ribosomal protein L16p/L10e family protein.	Involved in translational regulation
	AT1G08360	Cyt Ribosomal protein L1p/L10e family	Structural constituent of ribosome, RNA binding; Involved in translation, RNA processing
	AT1G04270.1, RIG	Encodes cytosolic ribosomal protein S15	Ribosome assembly
	AT1G01100	60S acidic ribosomal protein family	Plays an important role in the elongation step of protein synthesis
	AT1G07070	Ribosomal protein L35Ae family protein	Structural constituent of ribosome; Involved in translation, ribosome biogenesis
	AT2G24050.1, eIFiso4G2	MIF4G domain-containing protein / MA3 domain-containing protein	Plays a role in the accumulation of some potyvirus during viral infection
	AT3G60240.4, EIF4G	Eukaryotic translation initiation factor 4G	Component of the protein complex eIF4F, which is involved in the recognition of the mRNA cap,
	AT1G62410	MIF4G domain-containing protein	Translation initiation factor activity; Involved in translation, RNA metabolic process;
	AT5G10360.1, EMB3010	40S ribosomal protein S6-2	Play an important role in controlling cell growth and proliferation through the selective translation of particular classes of mRNA
	AT4G31700.1, RPS6A	40S ribosomal protein S6-1	Controls cell growth and proliferation through the selective translation of particular classes of mRNA

	AT3G08850.1, RAPTOR1	HEAT repeat ;WD domain, G-beta repeat protein	RAPTOR proteins are binding partners of the target of rapamycin kinase that is present in all eukaryotes
	AT3G10540	3-phosphoinositide-dependent protein kinase	Involved in protein amino acid phosphorylation;
	AT5G04510.1, PDK1	3'-phosphoinositide-dependent protein kinase 1	Couple lipid signals to the activation-loop phosphorylation of several protein kinases of the so-called AGC kinase family.
	AT1G50030.1, TOR	Serine/threonine-protein kinase TOR	Essential cell growth regulator that controls development from early embryo to seed production.
**RPL6 (AT1G05190.1), emb2394**	AT1G79850.1, RPS17	30S ribosomal protein S17, chloroplastic;	One of the primary rRNA binding proteins, it binds specifically to the 5'-end of 16S ribosomal RNA
	AT2G24090.1, PRPL35	50S ribosomal protein L35, chloroplastic;	Structural constituent of ribosome; Involved in translation; Located in ribosome, chloroplast;
	AT1G75350.1, emb2184	50S ribosomal protein L31, chloroplastic; Binds the 23S rRNA	
	AT1G74970.1, RPS9	Ribosomal protein S9	Component of the chloroplast ribosome
	AT1G32990.1, PRPL11	50S ribosomal protein L11, chloroplastic	Involved in Photosynthesis
	AT2G33800.1, EMB3113	30S ribosomal protein S5, chloroplastic	Binds directly to 16S ribosomal RNA
	AT3G44890.1, RPL9	50S ribosomal protein L9, chloroplastic;	Plastid ribosomal protein CL9
	AT1G07320.1, RPL4	50S ribosomal protein L4, chloroplastic	Binds directly and specifically to 23S rRNA and play a role in plastid transcriptional regulation, Belongs to the universal ribosomal protein uL4 family
	AT2G33450.1, PRPL28	50S ribosomal protein L28, chloroplastic	Structural constituent of ribosome
	AT5G40950.1, RPL27	50S ribosomal protein L27, chloroplastic	Structural constituent of ribosome; Involved in translation in thylakoid,

			ribosome, chloroplast stroma, chloroplast;
	AT1G78630.1, emb1473	50S ribosomal protein L13, chloroplastic	Structural constituent of ribosome; Involved in translation, embryo development ending in seed dormancy
	AT3G25920.1, RPL15	Encodes a plastid ribosomal protein CL15,	A constituent of the large subunit of the ribosomal complex; Belongs to the universal ribosomal protein uL15 family
	RPL27, AT5G40950.1	50S ribosomal protein L27 chloroplastic	Structural constituent of ribosome
	AT3G54210	50S ribosomal protein L17, chloroplastic	Directly binds 23S ribosomal RNA
	AT5G65220	50S ribosomal protein L29, chloroplastic	Structural constituent of ribosome, involved in translation, ribosome biogenesis
	AT2G43030	50S ribosomal protein L3-1, chloroplastic	One of the primary rRNA binding proteins, it binds directly near the 3'-end of the 23S rRNA, where it nucleates assembly of the 50S subunit
	AT1G35680.1, RPL21C	50S ribosomal protein L21, chloroplastic	Binds to 23S ribosomal RNA in the presence of protein L20
	AT3G54210	50S ribosomal protein L17, chloroplastic	Directly binds to 23S ribosomal RNA
	AT1G48350.1, EMB3105	50S ribosomal protein L18, chloroplastic	Binds 5S rRNA, forms part of the central protuberance of the 50S subunit
	AT3G15190.1, PRPS20	Chloroplast 30S ribosomal protein S20, putative	Binds directly to 16S ribosomal RNA; Belongs to the bacterial ribosomal protein bS20 family
	AT1G64510	Translation elongation factor EF1B/ribosomal protein S6 family protein	Binds together with S18 to 16S ribosomal RNA
	AT4G01310	50S ribosomal protein L5, chloroplastic	Binds 5S rRNA, forms part of the central protuberance of the 50S subunit
**RPL18 (AT3G05590.1),**	AT1G18080.1, ATARCA	Transducin/WD40 repeat-like superfamily protein	Major component of the RACK1 regulatory proteins that play a role in

			multiple signal transduction pathways.
	AT1G07070	Ribosomal protein L35Ae family protein	Structural constituent of ribosome
	AT1G15930	Ribosomal protein L7Ae/L30e/S12e/Gadd 45 family protein	Structural constituent of ribosome, involved in response to cadmium ion, response to salt stress, translation
	AT1G12960	Ribosomal protein L18e/L15 superfamily protein	Structural constituent of ribosome
	AT1G33120.1, RPL9B	Ribosomal protein L6 family	Structural constituent of ribosome, rRNA binding, involved in translation
	AT1G02780.1, emb2386	Ribosomal protein L19e family protein; Embryo defective 2386 (emb2386)	Structural constituent of ribosome, involved in translation, ribosome biogenesis, embryo development ending in seed dormancy
	AT1G14320.1, SAC52	Ribosomal protein L16p/L10e family protein	Involved in translational regulation. Contribute to general translation under UV-B stress. Involved in the NIK1- mediated defense response to geminivirus infection. Acts coordinately with LIMYB as a transcriptional repressor
	AT1G27400	Ribosomal protein L22p/L17e family protein	Structural constituent of ribosome, involved in translation
	AT1G15250	Zinc-binding ribosomal protein family protein	Binds to the 23S rRNA
	AT1G01100	60S acidic ribosomal protein family	Plays an important role in the elongation step of protein synthesis
	AT1G18540	Ribosomal protein L6 family protein	Structural constituent of ribosome
	AT1G09590	Translation protein SH3-like family protein	Structural constituent of ribosome
	AT1G23290.1, RPL27AB	Encodes a ribosomal protein L27A,	Regulated by TCP20, Belongs to the universal ribosomal protein uL15 family
	AT1G09590.1, RPL21A	Translation protein SH3-like family protein	Structural constituent of ribosome

	AT1G36240	Ribosomal protein L7Ae/L30e/S12e/Gadd 45 family protein	Ribosomal constituent
	AT1G08360	Ribosomal protein L1p/L10e family	Structural constituent of ribosome, involved in RNA binding; RNA processing; translation
	AT1G33140.1, PGY2	Encodes ribosomal protein L9	Identified in a screen for enhancers of as1. as1/pgy double mutants show defects in leaf vascular patterning and adaxial cell fate. Belongs to the universal ribosomal protein uL6 family
	AT1G41880	Ribosomal protein L35Ae family protein	Structural constituent of ribosome
	AT1G26910.1, RPL10B	Ribosomal protein L16p/L10e family protein	Structural constituent of ribosome, involved in response to UV-B, developmental process, translation
**RPL23.1 (ATCG00840.1)**	ATCG01230.1, RPS12B	30S ribosomal protein S12, chloroplastic	The gene is located in three distinct loci on the chloroplast genome and is transpliced to make one transcript
	ATCG00905.1, RPS12C	Chloroplast gene encoding ribosomal protein s12	The gene is located in three distinct loci on the chloroplast genome and is transpliced to make one transcript
	AT3G25520.1, RPL5A	60S ribosomal protein L5-1<br>:	Component of the ribosome, a large ribonucleoprotein complex responsible for the synthesis of proteins in the cell
	ATCG01310.1, RPL2.2	Encodes a chloroplast ribosomal protein L2	A constituent of the large subunit of the ribosomal complex, belongs to the universal ribosomal protein uL2 family
	AT3G60245	Zinc-binding ribosomal protein family protein	Structural constituent of ribosome
	RPL23.2	One of two chloroplast gene that encodes RPL23	A constituent of Large subunit of the ribosomal complex
**RPL24**	AT2G33450.1,	50S ribosomal protein	Structural constituent of

**(AT5G54600.1),**	PRPL28	L28, chloroplastic	ribosome
	AT1G32990.1, PRPL11	50S ribosomal protein L11, chloroplastic	Mutant has Decreased effective quantum yield of photosystem II; Pale green plants; Reduced growth rate
	AT3G44890.1, RPL9	50S ribosomal protein L9, chloroplastic	Plastid ribosomal protein CL9
	AT1G78630.1, emb1473	50S ribosomal protein L13, chloroplastic	Embryo defective 1473 (emb1473), structural constituent of ribosome embryo development ending in seed dormancy
	AT2G24090.1, PRPL35	50S ribosomal protein L35, chloroplastic	Structural constituent of ribosome
	AT3G25920.1, RPL15	Encodes a plastid ribosomal protein CL15	A constituent of the large subunit of the ribosomal complex
	AT3G54210	50S ribosomal protein L17, chloroplastic	Binds directly to 23S ribosomal RNA
	AT5G40950.1, RPL27	50S ribosomal protein L27, chloroplastic	Ribosomal protein large subunit 27 (RPL27) structural constituent of ribosome
	AT2G38140.1, PSRP4	30S ribosomal protein S31, chloroplastic	Plastid-specific ribosomal protein 4 (PSRP4) mRNA, complete, belongs to the bacterial ribosomal protein bTHX family
	AT1G79850.1, RPS17	30S ribosomal protein S17, chloroplastic	One of the primary rRNA binding proteins, it binds specifically to the 5'-end of 16S ribosomal RNA. Required for optimal plastid performance in terms of photosynthesis and growth. Required for the translation of plastid mRNAs. Plays a critical role in biosynthesis of thylakoid membrane proteins encoded by chloroplast genes
	AT1G74970.1, RPS9	Ribosomal protein S9,	A nuclear encoded component of the chloroplast ribosome

	AT1G07320.1, RPL4	50S ribosomal protein L4, chloroplastic	Binds directly and specifically to 23S rRNA). May play a role in plastid transcriptional regulation;
	AT1G35680.1, RPL21C	50S ribosomal protein L21, chloroplastic	Binds to 23S ribosomal RNA in the presence of protein L20
	AT2G43030	50S ribosomal protein L3-1, chloroplastic	One of the primary rRNA binding proteins, it binds directly near the 3'-end of the 23S rRNA, where it nucleates assembly of the 50S subunit
	AT1G05190.1, emb2394	50S ribosomal protein L6, chloroplastic	Binds directly to 23S ribosomal RNA and is located at the aminoacyl-tRNA binding site of the peptidyltransferase center
	AT2G33800.1, EMB3113	30S ribosomal protein S5, chloroplastic	Binds directly to 16S ribosomal RNA
	AT1G64510	Translation elongation factor EF1B/ribosomal protein S6 family protein	Binds together with S18 to 16S ribosomal RNA
	AT4G01310	50S ribosomal protein L5, chloroplastic	Binds 5S rRNA, forms part of the central protuberance of the 50S subunit
	AT3G15190.1, PRPS20	Chloroplast 30S ribosomal protein S20, putative	Binds directly to 16S ribosomal RNA; Belongs to the bacterial ribosomal protein bS20 family
	AT1G48350.1, EMB3105	50S ribosomal protein L18, chloroplastic	Binds 5S rRNA, forms part of the central protuberance of the 50S subunit
**RPS28 (AT5G64140.1)**	AT1G18080.1, ATARCA	Transducin/WD40 repeat-like superfamily protein	Major component of the RACK1 regulatory proteins that play a role in multiple signal transduction pathways
	AT1G04270.1, RIG	Encodes cytosolic ribosomal protein S15	Belongs to the universal ribosomal protein uS19 family
	AT1G15930	Ribosomal protein L7Ae/L30e/S12e/Gadd 45 family protein	Structural constituent of ribosome, involved in response to cadmium ion, response to salt stress, translation

	AT1G09590	Translation protein SH3-like family protein	Structural constituent of ribosome
	AT1G14320.1, SAC52	Ribosomal protein L16p/L10e family protein	Involved in translational regulation. Contribute to general translation under UV-B stress. Involved in the NIK1- mediated defense response to geminivirus infection. Acts coordinately with LIMYB as a transcriptional repressor
	AT1G02780.1, emb2386	Ribosomal protein L19e family protein; Embryo defective 2386 (emb2386);	Structural constituent of ribosome. Involved in translation, ribosome biogenesis, embryo development
	AT1G15250	Zinc-binding ribosomal protein family protein	Binds to the 23S rRNA
	AT1G08360	Ribosomal protein L1p/L10e family	Structural constituent of ribosome, RNA binding
	AT1G33140.1, PGY2	Encodes ribosomal protein L9	Identified in a screen for enhancers of as1. The as1/pgy double mutants show defects in leaf vascular patterning and adaxial cell fate. Double mutant analysis indicates pgy genes function in the same pathway as REV, KAN1 and KAN2
	AT1G27400	Ribosomal protein L22p/L17e family protein	Structural constituent of ribosome
	AT1G01100	60S acidic ribosomal protein family	Plays an important role in the elongation step of protein synthesis
	AT1G41880	Ribosomal protein L35Ae family protein	Structural constituent of ribosome, involved in translation, ribosome biogenesis
	AT1G09590.1, RPL21A	Translation protein SH3-like family protein	Structural constituent of ribosome
	AT1G12960	Ribosomal protein L18e/L15 superfamily protein	Structural constituent of ribosome
	AT1G18540	Ribosomal protein L6 family protein	Structural constituent of ribosome
	AT1G23290.1,	Encodes a ribosomal	A constituent of the large

	RPL27AB	protein L27A	subunit of the ribosomal complex. Regulated by TCP20; Belongs to the universal ribosomal protein uL15 family
	AT1G26880	Ribosomal protein L34e superfamily protein	Structural constituent of ribosome
	AT1G07070	Ribosomal protein L35Ae family protein	Structural constituent of ribosome
	AT1G33120.1, RPL9B	Ribosomal protein L6 family	Structural constituent of ribosome, rRNA binding
**RPS6**	AT1G09590.1, RPL21A	Translation protein SH3-like family protein	Structural constituent of ribosome
	AT1G14320.1, SAC52	Ribosomal protein L16p/L10e family protein	Involved in translational regulation. Contribute to general translation under UV-B stress. Involved in the NIK1- mediated defense response to geminivirus infection. Acts coordinately with LIMYB as a transcriptional repressor
	AT1G02830	Putative 60S ribosomal protein L22-1	Structural constituent of ribosome
	AT1G15250	Zinc-binding ribosomal protein family protein	Binds to the 23S rRNA
	AT1G15930	Ribosomal protein L7Ae/L30e/S12e/Gadd 45 family protein	Structural constituent of ribosome. Involved in response to cadmium ion, response to salt stress, translation
	AT1G02780.1, emb2386	Ribosomal protein L19e family protein; Embryo defective 2386 (emb2386)	Structural constituent of ribosome
	AT1G27400	Ribosomal protein L22p/L17e family protein	Structural constituent of ribosome
	AT1G26910.1, RPL10B	Ribosomal protein L16p/L10e family protein	Involved in response to UV-B, developmental process, translation
	AT1G08360	Ribosomal protein L1p/L10e family	Involved in RNA binding, translation, RNA processing
	AT1G18080.1, ATARCA	Transducin/WD40 repeat-like superfamily protein	Major component of the RACK1 regulatory proteins that play a role in

			multiple signal transduction pathways
	AT1G12960	Ribosomal protein L18e/L15 superfamily protein	Structural constituent of ribosome
	AT1G01100	60S acidic ribosomal protein family	Plays an important role in the elongation step of protein synthesis
	AT1G33140.1, PGY2	Encodes ribosomal protein L9	Identified in a screen for enhancers of as1. as1/pgy double mutants show defects in leaf vascular patterning and adaxial cell fate
	AT1G18540	Ribosomal protein L6 family protein	Structural constituent of ribosome
	AT1G36240	Ribosomal protein L7Ae/L30e/S12e/Gadd 45 family protein	Structural constituent of ribosome
	AT1G33120.1, RPL9B	Ribosomal protein L6 family	Structural constituent of ribosome, involved in rRNA binding
	AT1G23290.1, RPL27AB	Encodes a ribosomal protein L27A	A constituent of the large subunit of the ribosomal complex. Regulated by TCP20
	AT1G07070	Ribosomal protein L35Ae family protein	Structural constituent of ribosome
	AT1G26880	Ribosomal protein L34e superfamily protein	Structural constituent of ribosome
	AT1G04270.1, RIG	Encodes cytosolic ribosomal protein S15	Belongs to the universal ribosomal protein uS19 family
**Interaction between the TOR-RPS6A-RPS6B-RPL6-RPL18e-RPL23.1-RPL23.2-RPL24A-RPL24B-RPS28-S6K1-S6K2 proteins**	AT3G08850.1, RAPTOR1	HEAT repeat ;WD domain, G-beta repeat protein protein	RAPTOR proteins are binding partners of the target of rapamycin kinase that is present in all eukaryotes and play a central role in the stimulation of cell growth and metabolism in response to nutrients
	AT3G11400.2, EIF3G1	Eukaryotic translation initiation factor 3 subunit G	RNA-binding component of the eukaryotic translation initiation factor 3 (eIF-3) complex, which is involved in protein synthesis of a specialized repertoire of mRNAs and, together with other

			initiation factors, stimulates binding of mRNA and methionyl-tRNAi to the 40S ribosome
	AT1G18080.1, ATARCA	Transducin/WD40 repeat-like superfamily protein	Major component of the RACK1 regulatory proteins that play a role in multiple signal transduction pathways
	AT3G11940.1, RPS5A	One of two genes encoding the ribosomal protein S5	Mutants have semi-dominant developmental phenotypes
	AT1G56070.1, LOS1	Ribosomal protein S5/Elongation factor G/III/V family protein	Catalyzes the GTP-dependent ribosomal translocation step during translation elongation. During this step, the ribosome changes from the pre-translocational (PRE) to the post- translocational (POST) state as the newly formed A-site-bound peptidyl-tRNA and P-site-bound deacylated tRNA move to the P and E sites, respectively. Catalyzes the coordinated movement of the two tRNA molecules, the mRNA and conformational changes in the ribosome.
	AT1G18080.1, ATARCA	Transducin/WD40 repeat-like superfamily protein	Major component of the RACK1, involved in multiple hormone responses and developmental processes. MAPK cascade scaffolding protein involved in the protease IV and ArgC signaling pathway but not the flg22 pathway
	AT2G37270.2, RPS5B	One of two genes encoding the ribosomal protein S5	Expressed at a lower level compared to ATRPS5A
	AT1G09690.1, AT1G09590	Translation protein SH3-like family protein	Structural constituent of ribosome
	AT2G40290	Eukaryotic translation initiation factor 2	Functions in the early steps of protein synthesis

		subunit alpha homolog	by forming a ternary complex with GTP and initiator tRNA. This complex binds to a 40S ribosomal subunit, followed by mRNA binding to form a 43S pre-initiation complex. Junction of the 60S ribosomal subunit to form the 80S initiation complex is preceded by hydrolysis of the GTP bound to eIF-2 and release of an eIF-2-GDP binary complex.
	AT2G39390	Ribosomal L29 family protein	Structural constituent of ribosome
	AT1G61580.1, RPL3B	60S ribosomal protein L3-2, R-protein L3 B (RPL3B)	Structural constituent of ribosome
	AT1G33120.1, RPL9B	Ribosomal protein L6 family	Structural constituent of ribosome, rRNA binding
	AT2G42740.1, RPL16A	Ribosomal protein large subunit 16A	Component of the ribosome, a large ribonucleoprotein complex responsible for the synthesis of proteins in the cell
	AT3G04400.1, emb2171	Ribosomal protein L14p/L23e family protein, Embryo defective 2171 (emb2171);	Structural constituent of ribosome
	AT3G44890.1, RPL9	50S ribosomal protein L9, chloroplastic	Plastid ribosomal protein CL9
	AT3G47370	Ribosomal protein S10p/S20e family protein	Structural constituent of ribosome
	AT2G43030	50S ribosomal protein L3-1, chloroplastic	One of the primary rRNA binding proteins, it binds directly near the 3'-end of the 23S rRNA, where it nucleates assembly of the 50S subunit
	AT2G04390	Ribosomal S17 family protein	Structural constituent of ribosome
	AT2G05220	Ribosomal S17 family protein	Structural constituent of ribosome
	AT1G80750	Ribosomal protein L30/L7 family protein	Structural constituent of ribosome

	EMB 1080, AT3G48930.1	Nucleic acid binding, OB-fold-like protein	Structural constituent of ribosome
	AT1G43170.8, EMB220	Encodes a cytoplasmic ribosomal protein	Belongs to the universal ribosomal protein uL3 family<br>Identifier:
	AT1G48350.1, EMB3105	50S ribosomal protein L18, chloroplastic	Binds 5S rRNA, forms part of the central protuberance of the 50S subunit
	AT3G10610.1,	Ribosomal S17 family protein	Structural constituent of ribosome
	AT3G10950	Zinc-binding ribosomal protein family protein	Structural constituent of ribosome
	AT3G18740	Ribosomal protein L7Ae/L30e/S12e/Gadd 45 family protein	Structural constituent of ribosome
	AT3G16780	Ribosomal protein L19e family protein	Structural constituent of ribosome
	AT3G16080	Zinc-binding ribosomal protein family protein;	Structural constituent of ribosome. Binds to the 23S rRNA
	AT1G09590.1, RPL21A	Translation protein SH3-like family protein, RPL21e	Structural constituent of ribosome
	AT1G23290.1, RPL27AB	Encodes a ribosomal protein L27A	A constituent of the large subunit of the ribosomal complex. Regulated by TCP20
	AT3G25520.1, RPL5A	60S ribosomal protein L5-1	Component of the ribosome, a large ribonucleoprotein complex responsible for the synthesis of proteins in the cell
	AT2G39460.2, RPL23AA	Encodes a 60S ribosomal protein L23aA (AtrpL23aA).	Paralog of RLPL23aB
	AT1G69620.1, RPL34	Putative 60S ribosomal protein L34	Belongs to the eukaryotic ribosomal protein eL34 family
	AT1G29970.2, RPL18AA	60S ribosomal protein L18A-1 (RPL18AA);	Structural constituent of ribosome
	AT2G44860	Probable ribosome biogenesis protein RLP24	Involved in the biogenesis of the 60S ribosomal subunit. Ensures the docking of NOG1 to pre-

			60S particles
	AT1G57660	Translation protein SH3-like family protein	Structural constituent of ribosome
	AT1G08360	Ribosomal protein L1p/L10e family	Structural constituent of ribosome, RNA binding, RNA processing
	AT3G48960	Ribosomal protein L13e family protein	Structural constituent of ribosome
	AT1G36240	Ribosomal protein L7Ae/L30e/S12e/Gadd 45 family protein	Structural constituent of ribosome
	AT2G41840, Ribosomal protein S5 domain 2-type	Ribosomal protein S5 family protein	Structural constituent of ribosome
	AT1G64510	Translation elongation factor EF1B/ribosomal protein S6 family protein	Binds together with S18 to 16S ribosomal RNA
	AT1G02830	Putative 60S ribosomal protein L22-1/L22e	Structural constituent of ribosome
	AT2G20450	Ribosomal protein L14	Structural constituent of ribosome
	AT2G40590	Ribosomal protein S26e family protein	Structural constituent of ribosome
	AT1G48630.1, RACK1B_AT	Receptor for activated C kinase 1B, RACK1 subfamily	Encodes a protein with similarity to mammalian RACKs. RACKs function to shuttle activated protein kinase C to different subcellular sites and may also function as a scaffold through physical interactions with other proteins.
	AT2G19730	Ribosomal L28e protein family	Structural constituent of ribosome
	AT3G22230	Ribosomal L27e protein family	Structural constituent of ribosome
	AT3G44590	60S acidic ribosomal protein family	Plays an important role in the elongation step of protein synthesis
	AT2G40510	Ribosomal protein S26e family protein	Structural constituent of ribosome
	AT2G45710	Zinc-binding ribosomal protein family protein	Structural constituent of ribosome
	AT3G06680	Ribosomal L29e protein family	Structural constituent of ribosome
	AT1G48830	Ribosomal protein S7e family protein	Structural constituent of ribosome

	AT2G21580	Ribosomal protein S25 family protein	Structural constituent of ribosome
	AT3G09500	Ribosomal L29 family protein	Structural constituent of ribosome
	AT2G36160	Ribosomal protein S11 family protein	Structural constituent of ribosome
	AT1G14320.1, SAC52	Ribosomal protein L16p/L10e family protein	Contribute to general translation under UV-B stress. Involved in the NIK1- mediated defense response to geminivirus infection. Acts coordinately with LIMYB as a transcriptional repressor
	AT2G01250	Ribosomal protein L30/L7 family protein	Structural constituent of ribosome
	AT2G32060	Ribosomal protein L7Ae/L30e/S12e/Gadd 45 family protein	Structural constituent of ribosome
	AT3G04840	Ribosomal protein S3Ae	Structural constituent of ribosome
	AT2G37600	Ribosomal protein L36e family protein	Structural constituent of ribosome
	AT1G77940	Ribosomal protein L7Ae/L30e/S12e/Gadd 45 family protein	Structural constituent of ribosome
	AT3G28900	Ribosomal protein L34e superfamily protein	Structural constituent of ribosome
	AT1G52300	Zinc-binding ribosomal protein family protein	Binds to the 23S rRNA
	AT1G02780.1, emb2386	Ribosomal protein L19e family protein, Embryo defective 2386 (emb2386);	Involved in translation, ribosome biogenesis, embryo development ending in seed dormancy
	AT1G15250	Zinc-binding ribosomal protein family protein	Binds to the 23S rRNA
	AT1G26880	Ribosomal protein L34e superfamily protein	Structural constituent of ribosome
	AT2G09990	Ribosomal protein S5 domain 2-like superfamily protein	Structural constituent of ribosome
	AT2G47570	Ribosomal protein L18e/L15 superfamily protein	Structural constituent of ribosome
	AT3G07110	Ribosomal protein L13 family protein	Structural constituent of ribosome
	AT3G09630	Ribosomal protein	Structural constituent of

		L4/L1 family	ribosome
	AT2G37190	Ribosomal protein L11 family protein	Binds directly to 26S ribosomal RNA
	AT1G15930	Ribosomal protein L7Ae/L30e/S12e/Gadd 45 family protein	Structural constituent of ribosome
	AT3G24830	Ribosomal protein L13 family protein	Structural constituent of ribosome
	AT3G23390.1	Zinc-binding ribosomal protein family protein	Structural constituent of ribosome
	AT1G12960	Ribosomal protein L18e/L15 superfamily protein	Structural constituent of ribosome
	AT2G47610	Ribosomal protein L7Ae/L30e/S12e/Gadd 45 family protein	Structural constituent of ribosome
	AT3G06700	Ribosomal L29e protein family	Structural constituent of ribosome
	AT1G57860.1	Translation protein SH3-like family protein, RPL21e	Structural constituent of ribosome
	AT3G05560	Ribosomal L22e protein family	Structural constituent of ribosome
	AT1G66580.1, SAG24	Senescence associated gene 24 (SAG24)	Structural constituent of ribosome
	AT2G18020.1, EMB2296	Ribosomal protein L2 family, Embryo defective 2296 (EMB2296);	Structural constituent of ribosome
	AT2G27720	60S acidic ribosomal protein family	Structural constituent of ribosome
	AT1G18540	Ribosomal protein L6 family protein	Structural constituent of ribosome
	AT1G04270.1, RIG	Encodes cytosolic ribosomal protein S15	Belongs to the universal ribosomal protein uS19 family
	AT2G19740	Ribosomal protein L31e family protein	Structural constituent of ribosome
	AT3G02190	Ribosomal protein L39 family protein	Structural constituent of ribosome
	AT3G13580	Ribosomal protein L30/L7 family protein	Structural constituent of ribosome
	AT3G04770.1, RPSAb	40S ribosomal protein Sa-2	Required for the assembly and/or stability of the 40S ribosomal subunit. Required for the processing of the 20S

			rRNA- precursor to mature 18S rRNA in a late step of the maturation of 40S ribosomal subunits; Belongs to the universal ribosomal protein uS2 family
	AT1G74060	Ribosomal protein L6 family protein	Structural constituent of ribosome
	AT1G29965	Ribosomal protein L18ae/LX family protein	Structural constituent of ribosome
	AT2G25210	Ribosomal protein L39 family protein	Structural constituent of ribosome
	AT3G11250	Ribosomal protein L10 family protein	Ribosomal protein P0 is the functional equivalent of E.coli protein L10
	AT1G33140.1, PGY2	Encodes ribosomal protein L9	Identified in a screen for enhancers of as1. as1/pgy double mutants show defects in leaf vascular patterning and adaxial cell fate. Double mutant analysis indicates pgy genes function in the same pathway as REV, KAN1 and KAN2
	AT1G41880	Ribosomal protein L35Ae family protein	Structural constituent of ribosome
	AT1G74270	Ribosomal protein L35Ae family protein	Structural constituent of ribosome
	AT1G58380.1, XW6	Ribosomal protein S5 family protein, XW6	Structural constituent of ribosome
	AT2G44120	Ribosomal protein L30/L7 family protein	Structural constituent of ribosome
	AT3G02560	Ribosomal protein S7e family protein	Structural constituent of ribosome
	AT3G04920	Ribosomal protein S24e family protein	Structural constituent of ribosome
	AT2G31610	Ribosomal protein S3 family protein	Structural constituent of ribosome. Involved in response to salt stress, translation, response to abiotic stimulus
	AT3G09200	Ribosomal protein L10 family protein	Ribosomal protein P0 is the functional equivalent of E.coli protein L10
	AT2G32220	Ribosomal L27e protein	Structural constituent of

		family	ribosome
	AT2G17360	Ribosomal protein S4 (RPS4A) family protein	Structural constituent of ribosome
	AT2G27530.1, PGY1	Encodes ribosomal protein L10aP	Identified in a screen for enhancers of as1. as1/pgy double mutants show defects in leaf vascular patterning and adaxial cell fate. Double mutant analysis indicates pgy genes function in the same pathway as REV, KAN1 and KAN2
	AT1G01100	60S acidic ribosomal protein family	Plays an important role in the elongation step of protein synthesis
	AT2G47110.1, UBQ6	Ubiquitin-40S ribosomal protein S27a-2	Ubiquitin exists either covalently attached to another protein, or free (unanchored)
	AT2G40010	Ribosomal protein L10 family protein	Belongs to the universal ribosomal protein uL10 family
	AT1G70600	Ribosomal protein L18e/L15 superfamily protein	Structural constituent of ribosome
	AT2G34480	Ribosomal protein L18ae/LX family protein	Structural constituent of ribosome
	AT3G09680	Ribosomal protein S12/S23 family protein	Structural constituent of ribosome
	AT3G11510	Ribosomal protein S11 family protein	Structural constituent of ribosome
	AT3G02080	Ribosomal protein S19e family protein	Structural constituent of ribosome
	AT1G26910.1, RPL10B	Ribosomal protein L16p/L10e family protein	Structural constituent of ribosome
	AT3G04230	Ribosomal protein S5 domain 2-like superfamily protein	Structural constituent of ribosome
	AT3G49010.3, BBC1	Encodes 60S ribosomal protein L13	Homolog of human breast basic conserved 1 (BBC1)
	AT1G72370.1, P40	40S ribosomal protein Sa-1	Required for the assembly and/or stability of the 40S ribosomal subunit. Required for the processing of the 20S rRNA- precursor to mature

			18S rRNA in a late step of the maturation of 40S ribosomal subunits
	AT3G14600	Ribosomal protein L18ae/LX family protein	Involved in N-terminal protein myristoylation, translation, ribosome biogenesis
	AT2G27710	60S acidic ribosomal protein family. Belongs to the eukaryotic ribosomal protein P1/P2	Plays an important role in the elongation step of protein synthesis
	AT1G07070	Ribosomal protein L35Ae family protein	Involved in translation, ribosome biogenesis
	AT1G74050	Ribosomal protein L6 family protein	Structural constituent of ribosome
	AT1G27400	Ribosomal protein L22p/L17e family protein	Structural constituent of ribosome
	AT1G67430	Ribosomal protein L22p/L17e family protein	Structural constituent of ribosome

Table. S7. The TOR, RPS6A, RPS6B, RPL6, RPL18, RPL23.1, RPL23.2, RPL24A, RPL24B, RPS28, S6K1, and S6K2 enrichment analysis

**Statistical background is assumed: Biological process Gene Ontology**				
**number of nodes:** 115<br>**number of edges:** 5707<br>**avg. local clustering coefficient:** 0.971<br>**PPI enrichment p-value:** < 1.0x 10^{-16}				
**GO term**	**Description**	**Count in network**	**Strength**	**False discovery rate**
GO:0006407	rRNA export from nucleus	2 of 2	2.32	0.0011
GO:0002181	cytoplasmic translation	35 of 63	2.06	3.86E-56
GO:0000463	maturation of LSU-rRNA from tricistronic rRNA transcript (SSU-rRNA, 5.8S rRNA, LSU-rRNA)	6 of 12	2.02	1.87E-09
GO:0000461	endonucleolytic cleavage to generate mature 3'-end of SSU-rRNA from (SSU-rRNA, 5.8S rRNA, LSU-rRNA)	2 of 4	2.02	0.0026
GO:0000027	ribosomal large subunit assembly	16 of 33	2	1.19E-24
GO:0042273	ribosomal large subunit biogenesis	29 of 69	1.94	1.75E-43
GO:1902626	assembly of large subunit precursor of preribosome	2 of 5	1.92	0.0036
GO:0000447	endonucleolytic cleavage in ITS1 to	2 of 5	1.92	0.0036

	separate SSU-rRNA from 5.8S rRNA and LSU-rRNA from tricistronic rRNA transcript (SSU-rRNA, 5.8S rRNA, LSU-rRNA)			
GO:0000470	maturation of LSU-rRNA	9 of 23	1.91	2.80E-13
GO:0042255	ribosome assembly	29 of 79	1.88	4.10E-42
GO:0000028	ribosomal small subunit assembly	13 of 36	1.88	1.01E-18
GO:0042274	ribosomal small subunit biogenesis	19 of 62	1.8	3.80E-26
GO:0000462	maturation of SSU-rRNA from tricistronic rRNA transcript (SSU-rRNA, 5.8S rRNA, LSU-rRNA)	7 of 23	1.8	9.16E-10
GO:0006412	translation	120 of 433	1.76	3.47E-191
GO:0030490	maturation of SSU-rRNA	8 of 30	1.74	1.01E-10
GO:0022618	ribonucleoprotein complex assembly	29 of 122	1.69	2.00E-37
GO:0042254	ribosome biogenesis	56 of 250	1.67	2.01E-73
GO:0006414	translational elongation	5 of 33	1.5	9.76E-06
GO:0016072	rRNA metabolic process	21 of 142	1.49	3.93E-23
GO:0006364	rRNA processing	20 of 136	1.48	5.41E-22
GO:0010506	regulation of autophagy	2 of 14	1.47	0.0171
GO:0030307	positive regulation of cell growth	2 of 16	1.41	0.0211
GO:0009955	adaxial/abaxial pattern specification	4 of 34	1.39	0.0003
GO:0045727	positive regulation of translation	2 of 17	1.39	0.0229
GO:0043410	positive regulation of MAPK cascade	2 of 23	1.26	0.0364
GO:0001934	positive regulation of protein phosphorylation	4 of 48	1.24	0.00098
GO:0032270	positive regulation of cellular protein metabolic process	6 of 123	1.01	0.00034
GO:0010224	response to UV-B	3 of 63	1	0.0239
GO:0044267	cellular protein metabolic process	123 of 2826	0.96	3.07E-106
GO:0034645	cellular macromolecule biosynthetic process	121 of 2793	0.95	6.45E-103
GO:0044271	cellular nitrogen compound biosynthetic process	121 of 2881	0.94	1.78E-101
GO:0010467	gene expression	121 of 2929	0.93	1.13E-100
GO:0009735	response to cytokinin	8 of 212	0.89	0.00011
GO:0006417	regulation of translation	4 of 130	0.81	0.024
GO:0071840	cellular component organization or biogenesis	59 of 2471	0.7	1.03E-25
GO:0006996	organelle organization	30 of 1283	0.69	6.80E-12
GO:0009409	response to cold	7 of 347	0.62	0.0112
GO:0016043	cellular component organization	32 of 2271	0.47	2.85E-07
GO:0009987	cellular process	126 of 5356	0.39	7.66E-43

**Molecular function**	**Gene Ontology**			
**GO term**	**Description**	**Count in network**	**Strength**	**False discovery rate**
GO:0006407	rRNA export from nucleus	2 of 2	2.32	0.0011
GO:0002181	cytoplasmic translation	35 of 63	2.06	3.86E-56
GO:0000463	maturation of LSU-rRNA from tricistronic rRNA transcript (SSU-rRNA, 5.8S rRNA, LSU-rRNA)	6 of 12	2.02	1.87E-09
GO:0000461	endonucleolytic cleavage to generate mature 3'-end of SSU-rRNA from (SSU-rRNA, 5.8S rRNA, LSU-rRNA)	2 of 4	2.02	0.0026
GO:0000027	ribosomal large subunit assembly	16 of 33	2	1.19E-24
GO:0042273	ribosomal large subunit biogenesis	29 of 69	1.94	1.75E-43
GO:1902626	assembly of large subunit precursor of preribosome	2 of 5	1.92	0.0036
GO:0000447	maturation of LSU-rRNA	7 of 23	1.76	3.47E-191
GO:0000470	ribosome assembly	120 of 433	1.74	1.01E-10
GO:0042255	ribosomal small subunit assembly	8 of 30	1.69	2.00E-37
GO:0000028	ribosomal small subunit biogenesis	29 of 122	1.67	2.01E-73
GO:0042274	maturation of SSU-rRNA from tricistronic rRNA transcript (SSU-rRNA, 5.8S rRNA, LSU-rRNA)	56 of 250	1.5	9.76E-06
GO:0000462	translation	5 of 33	1.49	3.93E-23
GO:0006412	maturation of SSU-rRNA	21 of 142	1.48	5.41E-22
GO:0030490	ribonucleoprotein complex assembly	20 of 136	1.47	0.0171
GO:0022618	ribosome biogenesis	2 of 14	1.41	0.0211
GO:0042254	translational elongation	2 of 16	1.39	0.0003
GO:0006414	rRNA metabolic process	4 of 34	1.39	0.0229
GO:0016072	rRNA processing	2 of 17	1.26	0.0364
GO:0006364	regulation of autophagy	2 of 23	1.24	0.00098
GO:0010506	positive regulation of cell growth	4 of 48	1.01	0.00034
GO:0030307	adaxial/abaxial pattern specification	6 of 123	1	0.0239
GO:0009955	positive regulation of translation	3 of 63	0.96	3.07E-106
GO:0045727	positive regulation of MAPK cascade	123 of 2826	0.95	6.45E-103
GO:0043410	positive regulation of protein phosphorylation	121 of 2793	0.94	1.78E-101
GO:0001934	positive regulation of cellular protein metabolic process	121 of 2881	0.93	1.13E-100
GO:0032270	response to UV-B	121 of 2929	0.89	0.00011
GO:0010224	cellular protein metabolic process	8 of 212	0.81	0.024
GO:0044267	cellular macromolecule biosynthetic process	4 of 130	0.7	1.03E-25
GO:0034645	cellular nitrogen compound biosynthetic	59 of	0.69	6.80E-12

	process	2471		
GO:0044271	gene expression	30 of 1283	0.62	0.0112
GO:0010467	response to cytokinin	7 of 347	0.47	2.85E-07
GO:0009735	regulation of translation	32 of 2271	0.39	7.66E-43
